# Novel *Bacillus* and *Prestia* isolates from Dwarf century plant enhance crop yield and salinity tolerance

**DOI:** 10.1038/s41598-024-65632-x

**Published:** 2024-06-25

**Authors:** Sanjoy Kumar Dhar, Jaspreet Kaur, Gajendra Bahadur Singh, Arjun Chauhan, Jeewan Tamang, Nikita Lakhara, Lyudmila Asyakina, Victor Atuchin, Gaurav Mudgal, Gholamreza Abdi

**Affiliations:** 1 University Institute of Biotechnology, Chandigarh University, Mohali, Punjab 140413 India; 2 Department of Biotechnology, Institute of Applied Sciences & Humanities, GLA University, Mathura, Uttar Pradesh 281406 India; 3 University Institute of Agricultural Sciences, Chandigarh University, Mohali, Punjab 140413 India; 4 Khaniyabas Rural Municipality, Province 3, Dhading, Bagmati Zone 45100 Nepal; 5 Laboratory for Phytoremediation of Technogenically Disturbed Ecosystems, Kemerovo State University, Krasnaya Street, 6, Kemerovo, Russia 650000; 6 Laboratory of Optical Materials and Structures, Institute of Semiconductor Physics, SB RAS, Novosibirsk, Russia 630090; 7 Research and Development Department, Kemerovo State University, Kemerovo, Russia 650000; 8 Department of Industrial Machinery Design, Novosibirsk State Technical University, Novosibirsk, Russia 630073; 9 R&D Center “Advanced Electronic Technologies”, Tomsk State University, Tomsk, Russia 634034; 10 Center for Waste Management and Renewable Energy, Saveetha Dental College and Hospitals, Saveetha Institute of Medical and Technical Sciences, Saveetha University, Chennai, 600077 India; 11 Department of Biotechnology, Persian Gulf Research Institute, Persian Gulf University, Bushehr, 75169 Iran

**Keywords:** Agavaceae, Endophytes, Wheat, Salt tolerant bacteria, Seed treatment, Growth promotion, Salinity, Stress resilience, Plant biotechnology, Plant breeding, Plant cell biology, Plant physiology, Plant signalling, Plant stress responses, Plant symbiosis

## Abstract

Soil salinity is a major environmental stressor impacting global food production. Staple crops like wheat experience significant yield losses in saline environments. Bioprospecting for beneficial microbes associated with stress-resistant plants offers a promising strategy for sustainable agriculture. We isolated two novel endophytic bacteria, *Bacillus cereus* (ADJ1) and *Priestia aryabhattai* (ADJ6), from *Agave desmettiana* Jacobi. Both strains displayed potent plant growth-promoting (PGP) traits, such as producing high amounts of indole-3-acetic acid (9.46, 10.00 µgml^−1^), ammonia (64.67, 108.97 µmol ml^−1^), zinc solubilization (Index of 3.33, 4.22, respectively), ACC deaminase production and biofilm formation. ADJ6 additionally showed inorganic phosphate solubilization (PSI of 2.77), atmospheric nitrogen fixation, and hydrogen cyanide production. Wheat seeds primed with these endophytes exhibited enhanced germination, improved growth profiles, and significantly increased yields in field trials. Notably, both ADJ1 and ADJ6 tolerated high salinity (up to 1.03 M) and significantly improved wheat germination and seedling growth under saline stress, acting both independently and synergistically. This study reveals promising stress-tolerance traits within endophytic bacteria from *A. desmettiana*. Exploiting such under-explored plant microbiomes offers a sustainable approach to developing salt-tolerant crops, mitigating the impact of climate change-induced salinization on global food security.

## Introduction

Globally, the productivity of crops faces significant challenges involving both quality and quantity attributes, arising from a diverse range of influencers in the environment. Losses in staple cereal and tuber crops have profoundly impacted global food security as they accounted for over 60% of the world’s food. Of the many stressors, poor soil profiles with high salinity, pollutants, and other edaphic cues critically impact crop physiology, leading to their diminished or suboptimal growth^1^. The compounding effect of these, coupled with the socio-economic developments and the continually escalating global climatic shifts, contribute to substantially declining commercial crop losses^2,3^. Climate change-driven rising sea levels are also projected to exacerbate soil salinity intrusion into coastal croplands and irrigation systems worldwide^4,5^. Wheat, a major staple food crop witnesses surprising yield losses in the range of 25–65%^6^ hailing from soils where salinity and drought are accountably the more severe impactors^7–10^. In tackling this challenge, strategies such as traditional breeding techniques, the creation of genetically modified crops, silviculture, crop rotation, and mixed farming methods have been canonical^11,12^. Other than this, the next-generation research is exploring how both transcription factors and growth regulators can enhance plant stress resilience^13–15^. However, these approaches are variously constrained by either regulatory, technical, cost, time, and labor challenges or withstand environmental risks. More promiscuously, the use of plant growth-promoting (PGP) microbes, many of which can facilitate plant growth under adverse environmental conditions including soil salinity, has emerged as a promising approach due to its environmental safety, ease of application, sustainability, and cost-effectiveness offerings^10,16–20^. Although many inherent microbial partners of wheat itself have shown potential as biofertilizers, biocontrol, and pollutant bioremediation agents^21–26^, except a few^6,27,28^, their usability under high saline environments remains questionable as is largely untested. Striving to sustainably nourish a growing global population, research communities are also exploring translational research lines over the usability of resilient plant systems and their relationships with microorganisms^29–34^. Microbial inhabitants of stress-resilient plants probably offer more worthwhile solutions as many such microbes are known to mimic their resilient plant hosts into possessing salinity and other stress-countering features^31,35–37^.

Many plant members of the genus *Agave* are slow-growing xerophytic succulent perennials, which easily thrive in harsh environments of the arid and semi-arid regions characterized by low water availability, poor soil fertility, and often high salinity^38^. They exhibit unique physiological and morphological adaptations that make them intriguing subjects for biotechnological investigations^39–43^ and for catering to land reclamation and ecological restoration in degraded ecosystems^41,43–47^. The development of stress-resilient food crops is a pressing need in the face of changing climate patterns, and agave plants could provide valuable genetic material for breeding programs aiming to enhance water use efficiency and stress tolerance in major crops^42,48,49^. Biotechnological strategies that capitalize on agave's ability to hyperaccumulate salt and heavy metals, or assist in soil stabilization, could contribute to phytoremediation efforts, addressing soil degradation and pollution^50–52^. The intriguing scope also exists in exploring the microbial communities associated with agave plants, a few of which have documented roles in bolstering plant growth, improving nutrient uptake, and mitigating stress^34,53–61^.

The smooth, spineless jade agaves also best known as Dwarf Century plants, *Agave desmettiana* Jacobi (ADJ), are small, perennial succulents native to southeastern Mexico. They are compact, low-growing agaves that form a rosette of rigid, smooth, light green leaves, which unlike other agaves lack spines or teeth on the margins. The leaves can reach up to 2–3 feet long. The entire plant grows to about 5 feet tall and 8 feet wide when mature. This species is appreciated for its ornamental value in xeriscapes, and rock gardens in many regions beyond its nativity. Other than ornamental assets, ADJ plants have been very limitedly explored and remained underutilized for their stress resilience attributes and their inherent microbial communities. Hence, it was intriguing to ascertain whether ADJ plants harbor prospective PGP microbial partners and whether the latter would allow bioprospecting regimes to translate enhanced stress tolerance in crop plants, particularly mitigating the effects of high saline environments. In this investigation, a comprehensive characterization of plant growth promotion attributes of two novel *Bacilli* isolates from ADJ plants was conducted. Our focus extended as well to an in-depth assessment of their ability to confer enhanced salt tolerance in commercial wheat seedlings. This work stands out as the first-ever study on Plant Growth Promoting Bacteria (PGPBs) availed from ADJ plants and thereof as well as their translational prospects for significant productivity in a commercial farming system.

## Materials and methods

### Plant material processing and surface sterilization

The collection and cultivation of ADJ plants in the presented study strictly followed the guidelines and regulations at the locality and study site(s). Morphological features were analyzed as available to ensure accurate identification of the plants as ADJ and to differentiate them from others^62–64^. ADJ plant material was procured from a residential setting only after approved permission from the municipal council for the use of the material for educational and research purposes. Agave plants’ species identification was carried out by biodiversity conservation and taxonomical service experts at the biotechnology department of UIBT, Chandigarh University, Mohali, Punjab, India, where a voucher specimen (Deposit number: CUUIBT2024/Mudgal/ADJ01-04) has also been deposited in the publicly available Herbarium Collections unit. Leaf from a healthy ADJ plant was collected in June of 2023 from a rhizome cluster hailing from the crevice over a cemented sidewalk around a barren residential plot site at Shivalik City (Kharar, SAS Nagar Mohali, Punjab). The collected plant material underwent a surface sterilization procedure based on our previously established protocol for other succulent plants^35,65^ with slight modifications. In brief, the plant material was washed under running tap water for an hour. Subsequently, it was rinsed with Tween 20 foam for 2 min and then treated with a 0.5% Dettol solution. Later treatments were performed under a laminar airflow hood using a 0.1% mercuric chloride solution for 45 s, followed by a 70% ethanol treatment for 1 min. Finally, the material underwent three consecutive washes of 3 min each with sterile distilled water (SDW). After sterilization, leaf segments measuring 2–3 cm^2^ in size were excised and established onto 0.8% agar-supplemented Murashige and Skoog media (MS medium) containing 3% sucrose and various phytohormones. The culture media were adjusted to a final pH of 5.88 before autoclaving. The culture incubations were carried out under controlled conditions with a temperature of 22 ± 2 °C, relative humidity of 60–65%, and a photoperiod of 16 h light and 8 h dark.

### Isolation of bacterial endophytes

Following the above-surface sterilization procedures and in vitro establishment of ADJ explants, regular checks (as also with ADJ’s tissue culture regimes) were carried out for 4 weeks. During this time, any contamination that could be attributed to a source(s) other than the explants was excluded from the study, while those that (i) appeared after 4 weeks, and (ii) did not affect ADJ’s in vitro tissue growth, were screened for co-cultivating bacterial outgrowths on the media overlay. Bacterial colonies were further subcultured onto fresh nutrient agar (NA) plates. Single colonies from the NA plates were used to raise the starter culture in 5 ml of sterile nutrient broth (NB) following overnight incubations at 37 °C with agitation at 150 rpm in the dark. This step was undertaken to prepare glycerol stocks of cultures for further studies.

### Microscopy

*For recording morphologically distinguishing features, bacterial isolates were observed using a light microscope (Olympus CH20i). Plant materials that had been subjected to various treatments, both *in vitro* and ex vitro, with or without the presence of bacterial isolates were also analyzed using a Nikon-745 T stereomicroscope equipped with a 5-MP camera. These morphometrics assessments encompassed measurements of shoot, root, branch, and root hairs where necessary.* To further examine the structural details of the bacterial isolates, scanning electron microscopy (SEM) was conducted at UCRD, Chandigarh University. To prepare the samples, inoculum at the logarithmic growth phase was centrifuged (at 7000 rpm, 4 °C, 5 min) followed by two subsequent washes of the pellet with a 7.2 pH 1X PBS. Following the washes, the samples underwent overnight fixation at 4 °C in a solution containing 2.5% glutaraldehyde and then washed thrice using the phosphate buffer as before. The samples were further dehydrated in a stepwise manner using an ethanol gradient, ultimately reaching a final ethanol concentration of 100%. The dried pellet was sputter-coated with gold and subsequently analyzed using SEM^66^ with image recording at an accelerating voltage of 10 kV on a JEOL TouchScope series SEM Equipment (model JSM-IT500, JEOL, New Delhi, India).

### Biochemical characterization and enzyme activity profiling

To characterize the isolated bacteria, a comprehensive battery of biochemical assays was employed, including Gram staining and tests for methyl red, catalase, citrate utilization, indole production, Voges-Proskauer test, starch hydrolysis, urease activity, oxidase test, nitrate reduction, motility, hydrogen sulfide (H2S) production, tween hydrolysis, and utilization of various carbohydrate substrates (detailed in Supplementary Table 1). These assays adhere to the standardized approach for bacterial identification outlined in Bergey's Manual of Determinative Bacteriology^67,68^. In brief, cellulase activity was assessed by inoculating freshly grown cultures onto agar plates containing carboxymethyl cellulose (CMC). Following incubation at 37 °C for 24 h, the plates were flooded with iodine solution. The formation of a clear halo zone surrounding the bacterial growth indicated positive cellulase activity^69^. Proteolytic activity was evaluated by spot-inoculating bacteria onto skimmed milk agar plates (Himedia, India). After overnight incubation at 37 °C in darkness, the presence of a clear halo zone signified proteolytic activity^70^. Similar approaches were employed to assess lipase and pectinase activity. Briefly, bacteria were inoculated onto tributyrin agar base (for lipase activity) and Pectinase Screening Agar Medium (PSAM) (for pectinase activity), respectively. Incubation was carried out at 37 °C for 24–48 h (lipase) or 72 h (pectinase). The formation of a clear halo zone in either case indicated the corresponding enzymatic activity^71^. Finally, amylase activity was determined by spot-inoculating bacteria onto starch agar plates followed by incubation at 37 °C for 24–48 h. After incubation, the plates were flooded with 1% iodine solution for 20 min. The presence of a yellow halo zone surrounding the bacterial growth confirmed amylase activity on the starch agar^72^.

### Bacterial motility testing

To test bacterial motility, two established methods were utilized^73^. The first technique was the semi-solid agar method, in which bacteria were introduced into an agar butt (SIM Medium Butt; Himedia, Mumbai, India) by vertically stabbing the agar with a fine loop containing the bacterial culture. After overnight incubation at 28 °C, if the bacterial growth was seen spreading in the stabbed agar, it indicated positive motility. Other than this, bacterial motility was also inferred using light microscopy (Metzer, Vision plus-5000 DPCT) over a wet mount prepared over a glass slide.

### Antibiotic sensitivity assays

Antibiotic susceptibility testing was performed using the Kirby-Bauer disc diffusion method, following the guidelines outlined by the Clinical and Laboratory Standards Institute (CLSI)^74,75^. Overnight bacterial cultures were prepared by inoculating a single colony into Muller-Hinton broth (Himedia, Mumbai) and incubating at 28 °C. The turbidity of the cultures was adjusted to 0.5 McFarland standard (approximately 1.5 × 10^8^ CFU/mL) using sterile saline. A sterile cotton swab was dipped into the adjusted bacterial suspension and used to inoculate the entire surface of Muller-Hinton agar plates (Himedia, Mumbai) by streaking in three directions to obtain a uniform lawn of bacterial growth. After allowing the inoculated plates to dry for 3–5 min, antibiotic susceptibility discs (Himedia, Mumbai, India) were aseptically placed onto the agar surface using sterile forceps. The discs were spaced sufficiently apart to prevent overlapping of the inhibition zones. The plates were incubated at 28 °C for 16–18 h. Following incubation, the diameter of the inhibition zones around each antibiotic disc was measured in millimeters. The measurements were performed in triplicate, and the mean inhibition zone diameter was calculated for each antibiotic tested against each bacterial isolate. Based on the observed inhibition zone diameters, the isolates were classified as susceptible, intermediate, or resistant to each antibiotic according to the CLSI interpretive criteria^75^. One antibiotic to which the isolates exhibited susceptibility was selected for maintaining cryopreserved stocks and ensuring aseptic conditions during subsequent in vitro experiments.

### Molecular characterization of ADJ isolates

Genomic DNA was isolated from the bacterial isolates using a kit from Himedia, India. The 16S rRNA gene was PCR amplified using universal primers 27F (AGAGTTTGATCMTGGCTCAG) and 1492R (TACGGYTACCTT-GTTACGACTT). The PCR reaction contained PCR buffer, MgCl2, primers, dNTPs, Taq polymerase, and water. PCR program consisted of an initial denaturation (95 °C for 5 min), intermittent 32 cycles (of denaturation at 95 °C for 1 min, annealing at 54 °C for 1.5 min, and extension at 72 °C for 1 min), and a final extension (72 °C for 10 min). Amplicons were extracted, ligated into pGEM-T vector (Promega, New Delhi), and heat-shock transformed into DH5α bacteria. Recombinants were screened by blue-white selection and colony PCR. Plasmids were sequenced using M13 primers. Sequences were trimmed for vector regions and analyzed using VecScreen (https://www.ncbi.nlm.nih.gov/tools/vecscreen/), CHROMAS (version 2.6.6, Technelysium Pty Ltd), BLASTn (https://www.ncbi.nlm.nih.gov/genbank/) and EzBioCloud (https://www.ezbiocloud.net/), and aligned with ClustalW. MEGA (version 11.0.11, https://www.megasoftware.net/downloads/dload_win_gui) was used to construct phylogenetic trees^76^. All kit-related procedures strictly adhered to the manufacturer's recommendations. In addition to house-molecular cloning-led identification, the results were validated with the microbial identification service at MTCC, CSIR-IMTECH, Chandigarh (Punjab, India).

### PGP assays over ADJ isolates

Various standard assays used for assessing plant growth-promoting (PGP) properties of the ADJ isolates are as below:

#### Potassium solubilization

Evaluation of the isolates’ potassium solubilization ability used a standard spot plate assay on Aleksandrow agar media (Cat# M1996, Himedia, Mumbai, India)^77^. Briefly, a loopful of overnight-grow bacterial starter culture (raised in NB 37 °C; agitation at 150 rpm, dark) was inoculated onto this agar and incubated at 28 °C. Clear halo zones surrounding the bacterial growth indicated the positive ability of the culture isolates to solubilize inorganic potassium salt (potassium alumino silicate) in this media.

#### Phosphate solubilization

To assess phosphate solubilization by the bacterial isolates, a standard plate-based protocol^78^ was used with the media containing an insoluble source of phosphate (tricalcium phosphate). In brief, spot inoculation on this semisolid Pikovskaya's medium (Mumbai, India) if develops a clear zone or halo around the bacterial lawn, infers positive phosphate solubilization ability of the bacteria. The degree of solubilization can be quantitatively evaluated using the following formula:

$${\text{Phosphate }}\;{\text{solubilization}}\;{\text{index}} \left( {{\text{cm}}} \right) = \frac{{{\text{Colony}}\;{\text{diameter}} \left( {{\text{cm}}} \right) + {\text{Halozone}}\;{\text{diameter}}\left( {{\text{cm}}} \right)}}{{{\text{Colony}}\;{\text{diameter}} \left( {{\text{cm}}} \right)}}$$

#### ACC deaminase activity

Many PGPBs are known to possess the enzyme 1-aminocyclopropane-1-carboxylate (ACC) deaminase, the latter which plays a crucial role in alleviating the detrimental impacts caused by stress-induced ethylene production in plants. To screen for this activity in our ADJ isolates, respective inocula were checked for their culturability within 72 h (at 28 °C, dark) over Dworkin and Foster’s (DF) minimal salt media, admixed with 2 g/l (NH_4_)_2_SO_4_ as the sole nitrogen source and appropriate selection antibiotic^79^. Culturability on DF media suggests the endogenous ACC deaminase activity in tested bacteria.

#### Siderophore production

Siderophore production by the bacterial isolates was assessed using the Chrome Azurol S (CAS) assay. To eliminate residual iron traces, the test vessels were rinsed overnight with 3 M HCl, followed by thorough washing with sterile distilled water, as per standard protocol^80^. The CAS agar plate assay method^81^ was employed, where the bacterial isolates were streak-plated on nutrient agar supplemented with 10% CAS reagent and incubated at 28 °C for one week. The development of a yellow-orange halo around the bacterial growth on the plates indicated positive siderophore production.

#### IAA production

A standard protocol^82^ was used to test for indole-3-acetic acid (IAA) production. The isolates were grown in separate experimental setups in nutrient broth containing 0.1% tryptophan (28 °C, 120 rpm for 10 days). Following every 2-day intervals, 1 ml aliquots of centrifuged supernatants were withdrawn, to which 2 ml of Salkowski reagent (0.5 M ferric chloride in perchloric acid)^83^ and incubated in the dark for 30 min. The development of a pink color indicated IAA production. IAA concentration was quantified by measuring absorbance at 530 nm compared to a standard curve of commercial IAA.

#### Ammonia production

To explore ammonia production, a well-established procedure^67^ was followed in which bacterial growth was allowed in peptone water for 10 days (28 °C, 120 rpm). Intermittently, 1 ml of clarified culture supernatants (after centrifuging at 10,000 rpm, 15 min) were mixed with 50 µL of Nessler's reagent. Yellowish brown furfuryl that positively develops can be measured using spectrophotometry (OD_450nm_) to quantify ammonia released against ammonium sulfate used as a reference standard.

#### Zinc solubilization assay

This used a plate-based assay^84^, wherein the bacteria were spot-inoculated onto an agar medium containing 0.1% zinc oxide and then incubated at 28 °C for one week. Any clear zone formation around the colonies indicated positive zinc solubilization activity in the tested bacteria.

#### N2 fixation

To evaluate the N_2_-fixing potentials, a week-long growth of bacteria inoculated over an N_2_-free Jensen media^85^ was investigated. A relatively shiny bacterial streak infers positive nitrogen fixation by the tested bacteria.

#### Hydrogen cyanide production

In a standard assay^86^, bacteria were inoculated on nutrient agar (NA) plates supplemented with 4.4 g/L glycine. Following this, a sterile Whatman filter paper (No. 1) disc soaked in a 2% sodium carbonate solution prepared in 0.5% picric acid was placed near the bacterial lawn. This preparation followed dark incubations (at 28 °C) for 4–5 days would reveal positive hydrogen cyanide gas production resulting in an orange-to-red coloration on the filter paper.

#### Biofilm formation

To estimate biofilming attributes in the tested bacteria Congo red agar (CRA) plate-based assay was employed^87^. In brief, a 1.5% agar-supplemented brain heart infusion medium (37 g) was mixed with Congo red (0.8 g) and sucrose (50 g)(pH 7.2). Inoculation of the bacterial test cultures over the plates and overnight incubations (at 28 °C), if result in a blackening of the newly developed colonies and those with dry consistency would infer positive biofilming properties of the tested bacteria.

### Seed priming treatments with ADJ isolates

Wheat seeds (UNNAT PBW 343) procured from a local seed shop (in Kharar, Mohali, Punjab, India) were gently washed for 2 min in a mild froth made with 0.5 ml Tween-20 detergent in 50 ml tap water. Further cleaning rinses with generous amounts of sterile distilled water (SDW) over approximately half an hour were exercised. Following this, the seeds were soaked in autoclaved sterile tap water overnight for imbibition. The next day, they were administered with bacterial priming treatments. Under in vitro trials, clarified-cell-free spent culture supernatants obtained from overnight grown bacterial inocula (in NB medium at 37 °C, 150 rpm, dark) were used for wheat seed pretreatments. In brief, 10 g of seeds were soaked overnight in 50 ml of spent supernatant from raised from each bacterial isolate. The spent supernatants in all cases were freshly supplemented with ampicillin. Control treatments of seeds involved soaking under fresh NB. For the *ex vitro* and field trial regimes, seeds were primed overnight with each isolate in independent experiments with a seeding density of 1 × 10^8^ CFU ml^−1^. These inocula hailed from single colonies on NA and subsequently subcultured overnight in NB (at 37 °C, 150 rpm, dark). The bacteria-wheat seed cocultivation conditions were maintained at 37 °C, 50 rpm, and darkness. The control seed sets received treatment solely with NB media. Bacterial treatments in the field similarly involved scale-up preparations to 0.5 L in each case. All growth media were supplemented with ampicillin at a concentration of 34 mg/L as a selection pressure.

### Validation parameters for PGP effects over commercial wheat

To validate the PGP effects of ADJ-isolated endophytes, various in vitro, ex vitro, and field experiments were trialed. The in vitro trials were carried out in sealed jam jars, each with three seeds established over 50 ml of agar-supplemented MS media. Like these, trials included seed sets from various pretreatments with or without bacterial supernatants (discussed above). These jam jars were provided and kept under PTC room conditions and were observed routinely for seedling emergence, and other growth parameters for both the primed-treated and control-treated seed lots. All trials were repeated thrice each of which consisted of five replicates. Ex vitro trials employed a completely randomized design (CRD) set up in a glasshouse containment facility (conditioned at 25–27 °C temperature, 70–90% relative humidity, with natural day/night photoperiod shifts). In essence, both bacteria-primed and control-treated seeds were established over 4 cm deep soil beds contained within plastic trays (43 × 34 × 7 cm). Garden soil used in these trials was preciously mesh sorted, heat sterilized, and not pretreated with any biocides or fertilizers. All soil beds with seeds were initially watered by spray maneuvers on the same day post-sowing, using approximately 250 ml of sterilized tap water per tray. Subsequently, a consistent daily spray-watering regimen of 100 ml per tray was maintained. Once at every weekly interval, watering treatments were replaced with a booster dosing of each of the soil beds with a fresh overnight preparation (1 × 10^8^ CFU per ml) of respective bacterial isolate raised on NB, using the same spraying technique. Control beds were treated with sterile NB without bacterial isolates. Regular monitoring and recording of seedling growth profiles were conducted. This as well, as at specific time points, is followed with comprehensive assessments of various physical and physiological growth parameters. To substantially evaluate the efficacy of bacterization-assisted PGP benefits and, more significantly, to assess its impact on enhancing wheat productivity in controlled field trials were implemented. As before, these trials as well adhered to a Completely Randomized Design (CRD) set up in a polyhouse facility at the university farms. While all soil preparation, seeding, watering, and evaluation regimes were kept consistent like the glasshouse trials, in the case herewith the field setting, the bacterized and control-treated seeds were sown over approximately 9-inch-raised soil beds. The irrigation and booster dosing reparations were maintained as before with upscaled volumes except that they used a soil-drenching method instead of the spraying technique. Seed germination recordings were conducted at every two days intervals, and measurements of seedling growth commenced every week. In one consolidated effort, following the complete inherent drying of shoots across all replicates, the harvest data for wheat was recorded. To maintain consistency across all experimental sets, any observed weed outgrowths in the field plots were meticulously removed by hand.

### Salinity tolerance testing over ADJ endophytes

Testing the ability of ADJ isolates to grow over a range of salt (NaCl) concentrations (1–8%), involved observations of growth in both liquid (NB) and agar-supplemented semisolid bacteriological (NA) media (37 °C, dark, with or without shaking at 150 rpm, respectively). In independent assays respective to each of the ADJ endophyte isolates, inoculums were enriched to grow on high salt by employing a subculturing sequence, intermittently increasing the salinity percentage at every subculture, and selecting single colonies showing satisfactory growth for seeding the next higher salt media preparation. Ampicillin was added in both media types to ensure selection. Growth was ascertained using visual observation of the bacterial lawn on semi-solid media at 18 h post-inoculation. For a more detailed view, microscopic observations at the periphery of the bacterial colonies were also recorded. Growth in liquid suspension media was ascertained spectrophotometrically (OD_600nm_).

### Testing salinity tolerance of PGPB-primed wheat seeds

Wheat seeds were pretreated with or without each of the ADJ isolates and/or their mixed culture (inoculum preparation and seed treatments were conditioned similarly as before) and established ex vitro over Petri dishes containing various salt concentration profiles (50–200 mM) in separate tests in a trial. All experimental trials were carried out in PTC room conditions. Seed germination and growth profiles were recorded at periodic intervals and measures of physiological growth were evaluated at the end of 3 weeks’ trial duration.

### Plant physiological growth assays

The evaluation of phenolic compounds, flavonoids, and photosynthetic pigments present in the examined plant leaf samples followed standardized protocols^88–90^. In brief, assaying contents of chlorophylls and carotenoids, a gram leaf sample was homogenized with 20 ml of 80% acetone mixed with 0.5 mg of magnesium carbonate powder. After incubation of this preparation at 4 °C for an hour, 1 ml of a centrifugally derived (5000 rpm, 5 min) supernatant was further treated with 9 ml acetone. This preparation was processed for various spectrophotometric estimations (OD_663nm_, OD_645nm_, and OD_450nm_) of pigments, respectively. For the spectrophotometric determination of the total phenolic content (TPC), another 1 ml aliquot of supernatant was mixed with 5 ml Folin Cicocalteu reagent, and 2 ml 1 M sodium carbonate. as added. Following a 2-h incubation period in the dark, the dark blue colored reaction product was read spectrophotometrically (OD_760nm_). Readings were plotted over a gallic acid standard, and TPC results were expressed as gallic acid equivalents (GAE) of the sample. Similarly, the estimation of total flavonoid content (TFC) used 1 ml supernatant from the tissue homogenate mixed with 3 ml methanol, 0.2 ml of 10% AlCl_3,_ and 0.2 ml 1 M sodium acetate, finally making up the volume to 10 ml with SDW. Following half an hour of incubation in the dark, spectrophotometry was done for OD_430nm_. Quercetin served as a reference standard and the TFC values were expressed in quercetin equivalents (QE) of the sample.

### Statistical and computational approaches

All experiments described herewith were conducted with a minimum of three iterations, and each iteration comprised three or more repetitions. Graphs represented data values and standard errors, illustrating the mean and standard deviations from replicates across three or more trials. For barcoding analyses, widely utilized various software suites, including VECSCREEN (https://www.ncbi.nlm.nih.gov/tools/vecscreen/), DECIPHER (version 2.19.2)^91^, CLC Workbench (version 6.5.1). The 16S rRNA-based molecular characterization of ADJ1 and ADJ6 endophytes involved similarity searches in NCBI’s GenBank (https://www.ncbi.nlm.nih.gov/genbank/) and EzBioCloud (https://www.ezbiocloud.net) databases. Figures were created using MS Excel and MS PowerPoint.

## Results

### Bacterial endophyte isolates from leaf tissues of the Dwarf Century plant

**Figure 1:**
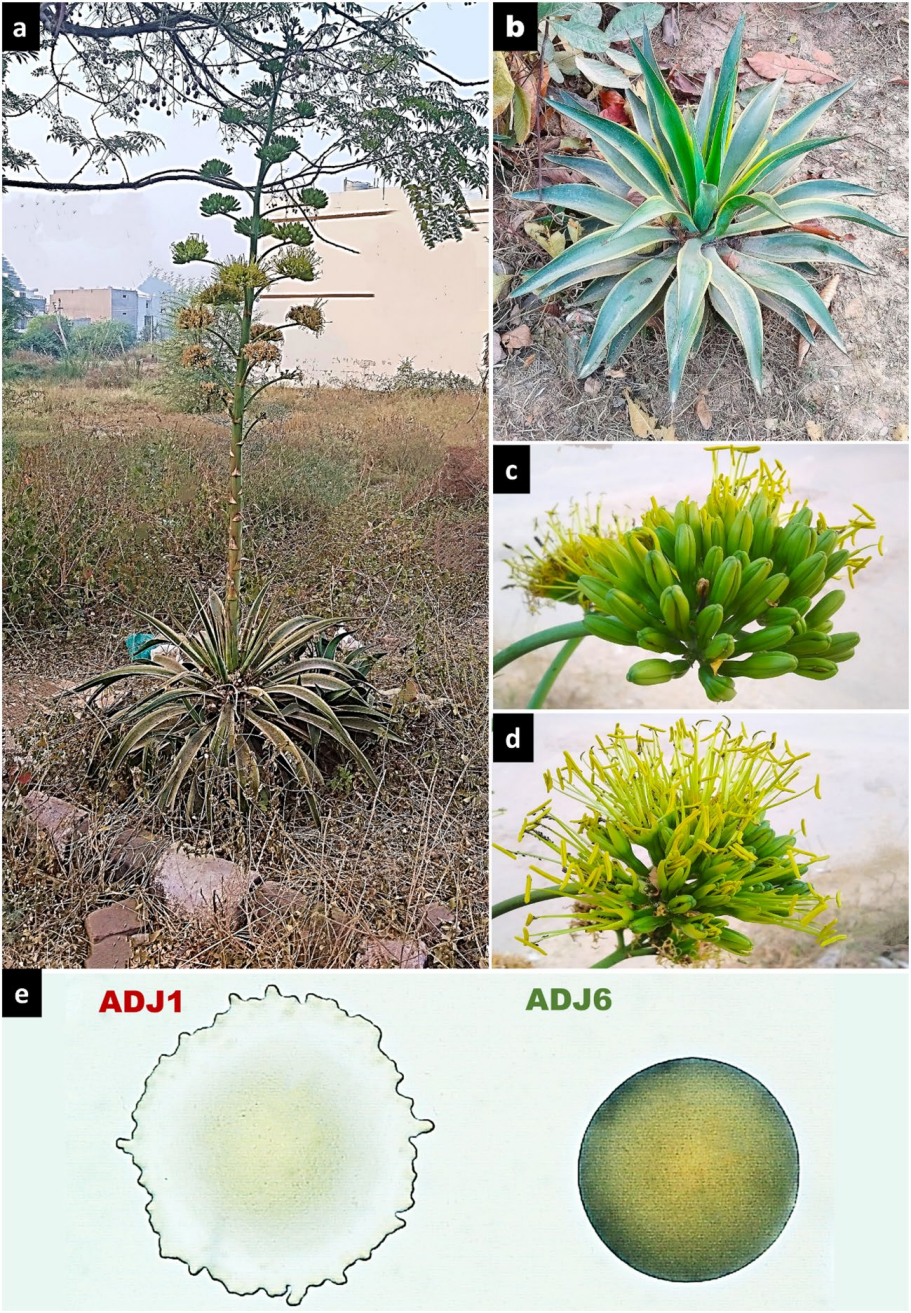
The dwarf Century plant, *A. desmettiana*, and its microbial isolates. Source ADJ plants wherein (**a**), residential plot, with a flowering stalk reaching up to 2.5 m and in (**b**), a clonal rhizomatic cluster of offsets in the vicinity of the former; in (**c**), closed flower buds (see texts for floral characteristics identifying it as ADJ); in (**d**), opened flower buds each with anthers surrounding an ovular stalk; in (**e**), light microscopy observations of separately growing ADJ1 and ADJ6 bacterial colonies, respectively with the swarm and smooth morphotypes as seen over NA plate following overnight incubation.

ADJ leaves were collected from a flowering cluster growing within an open residential plot site at Shivalik City (Kharar, SAS Nagar Mohali, Punjab) (Fig. 1a). It could be seen as well surrounded by its progeny clonal propagules in its close vicinity (Fig. 1b). The plant was identified as a variegated variety based on its peculiar floral characteristics (Fig. 1a-d)^92,93^. Bacteria were derived from the leaves in the lowermost offsets of the flowering cluster and studied later for their prospects as plant growth facilitators. Of the six endophyte isolates retrieved from serial dilution trials over the 1XPBS crushed lysate of the surface sterilized agave leaf segments, two potential plant growth promoting (PGP) types (named, ADJ1 and ADJ6) were selected, based on their masking culturability over other four isolates under NB inoculations, antibiotic sensitivity profiles, and high potentials under both qualitative and quantitative PGP assay parameters. Through close examination with light microscopy, it was speculated that ADJ1 and ADJ6 were developing as a combined co-culture involving two potential bacterial colonizers with distinct morphologies (Fig. 1e). One displayed rapidly expanding swarms with irregularly shaped colonies (ADJ1), while the other presented a smooth texture and rounded colonies (ADJ6). Notably, both ADJ1 and ADJ6 isolates appeared to grow harmoniously without any discernible zones of antagonism or inhibition (Fig. 1e).

### Morphological and biochemical profiles of ADJ1 and ADJ6

Morphological observations and results obtained in biochemical assays performed over the two ADJ isolates have been outlined in Supplementary Table 1. These results suggest that both ADJ1 and ADJ6 are rod-shaped, motile, gram-positive bacteria. The isolates showed profound activities for catalase, urease, oxidase, citrate utilization, and H_2_S production. However, only ADJ6 exclusively showed positive tests for methyl red, Voges Proskauer, indole, and starch hydrolysis, and also it could very well ferment glucose, sucrose, starch, lactose, dextrose, and gelatin. Both isolates showed negative activities for protease and lipase. SEM depicted structural distinguishments of the two ADJ-endophytes. Although both appeared as rod-shaped bacteria (Supplementary Fig. 1), their morphology drastically differed. ADJ1 looked more like biscuit pellets, while ADJ6 had a more solenoid appearance.

### Response to Antibiotics sensitivity assays of ADJ1 and ADJ6 isolates

Isolates ADJ1 and ADJ6 exhibited comparable antibiotic sensitivity, showing growth susceptibility to the majority of tested antibiotics. Both isolates share resistivity to ampicillin. however, ADJ1 displayed resistance exclusively to penicillin and cefepime (Supplementary Table 2), otherwise fair similarities in susceptibility to streptomycin, tobramycin, trimethoprim, chloramphenicol, clarithromycin, neomycin, and ofloxacin. Based on these studies, bacterial stocks were secured from contamination under culturing revivals from cryostorage as well as during various in vitro tests using the right selection agent in the bacteriological media.

### Molecular characterization of ADJ endophytes

**Figure 2:**
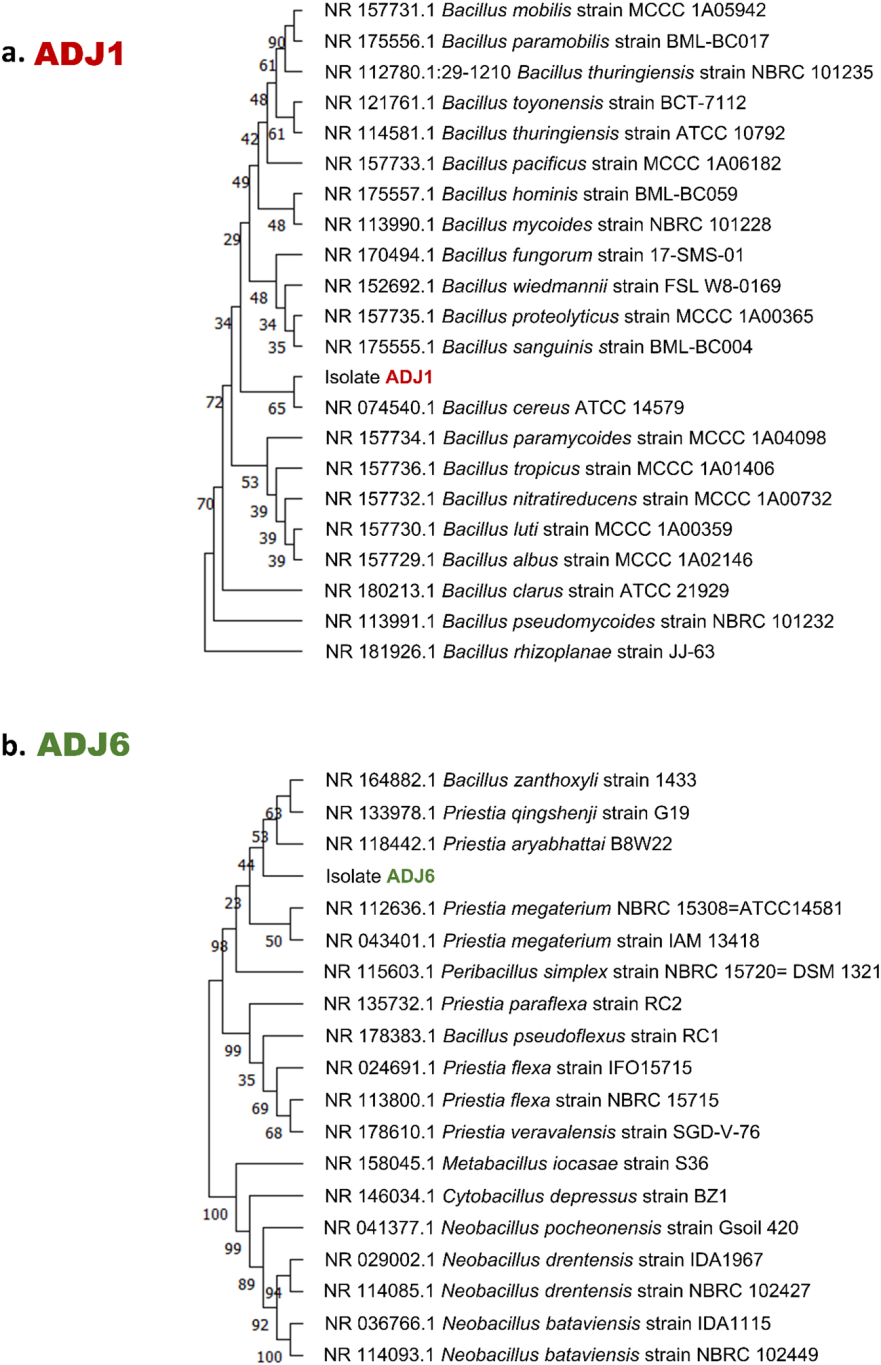
Phylogenetic relatedness with ADJ1 and ADJ6. Phylogenetic tree constructed using neighbor-joining algorithm over partial 16S rRNA gene sequences of (**a**), strain ADJ1 (1181 bases; GenBank accession OR898198), and (**b**), strain ADJ6 (1462 bases; GenBank accession OR898199) depicts the evolutionary relationship with other bacterial strains. The bootstrap values shown on the branches of the diagram were calculated from 1000 resampled datasets.

Neighbor-joining trees indicated a close relatedness amongst these two distinct species, situating them within the Bacillus genus (Fig. 2a,b), this was availed following the phylogenetic analyses utilizing the partial 16S rRNA gene sequences from isolates ADJ1 (1181 bases; GenBank accession number OR898198) and isolate ADJ6 (1462 bases; GenBank accession numbers OR898199). However, in context to the tree depictions, ADJ1 matched more closely to *Bacillus* species, while ADJ6 sat more closely amongst the taxonomically diverged variant of the *Bacillus*, the *Priestia* sp. Each of the isolates shares a common ancestor (Fig. 2a,b). The outcomes of the sequence similarity search additionally supported these observations, revealing that isolate ADJ1 exhibited a significantly higher level of identity in its 16S rRNA fragment, reaching an identity of 99.83% with *Bacillus cereus* ATCC 14579 strain, and this is at a 100% query coverage, while the other isolate ADJ6 with *Priestia aryabhattai* B8W22 of that of 99.85% sequence identity, however this latter at a possible query coverage of only 88%, respectively. Other than this, at the next slightly lower sequence identity score of 99.75% the ADJ1 isolate also showed sequence characters similar to seven other markedly different *Bacillus* sp., namely, *B. wiedmannii* (strain FSL W8-0169), *B. tropicus* (strain MCCC 1A01406), *B. proteolyticus* (strain MCCC 1A00365), *B. nitratireducens* (strain MCCC 1A00732), *B. luti* (strain MCCC 1A00359), *B. albus* (strain MCCC 1A02146), and *B. sanguinis* (strain BML-BC004 16S). The ADJ6 isolate with higher sequence coverage (97–99%), but then simultaneously with the next slightly lower identity scores (below 99.75–99.38%), showed nearness with four species in closely related genera: *Priestia, Bacillus,* and *Peribacillus* (percentage identity depicted respectively in braces with strain names ahead)*,* namely, *Priestia megaterium* (strain NBRC 15308 = ATCC 14581; 99.66%), *Peribacillus acanthi* (strain L28; 99.65%), *B. zanthoxyli* (strain 1433; 99.51%), and *Priestia megaterium* (strain IAM 13,418; 99.38%). Apart from the neighbor-joining algorithm (Fig. 2a,b), alternative phylogenetics generation algorithms, including maximum-parsimony, UPGMA, and maximum-likelihood produced congruent patterns in the resulting phylogenetic trees, thus supporting that ADJ1 is a novel *B. cereus* strain, while ADJ6 is a *P. aryabhattai* strain. Despite their molecular phylogenetic relatedness, both the isolates, ADJ1 and ADJ6 exhibited marked differences in colony and cell-specific morphology, biochemical characteristics, and antibiotic profiles. Moreover, this notion is also backed by their marked variations in assayed PGP properties and concomitantly evidenced effects on plant seedling growth and productivity.

### Plant growth promoting potentials of ADJ1 and ADJ6

As the bacteria were isolated from ADJ plant leaves, it was intriguing to assess which plant growth-boosting capabilities the isolates displayed. These PGP properties of both isolates are summarized in Table 1. Both isolates demonstrated the ability to produce ammonia, fix nitrogen, biosynthesize an auxin indole-3-acetic acid (IAA), and generate hydrogen cyanide gas. However, the analyses showed that neither ADJ1 nor ADJ6 isolates demonstrated the ability to solubilize potassium or produce siderophores. Both isolates tested negative for these two particular plant growth-promoting activities. In contrast, they displayed a relatively high extent of zinc solubilization and biofilm formation ability as well.

**Table 1:** Various plant growth promotion assays conducted with the ADJ1 and ADJ6 isolates.

*PGP attributes*	ADJ-1	ADJ-6
Phosphate solubilization index (cm)	−	2.77 ± 0.25
Siderophore production (%)	−	−
Ammonia production (µmol ml^−1^)	64.67 ± 0.50	108.97 ± 0.53
IAA production (µg ml^−1^)	9.46 ± 0.20	10.09 ± 0.10
Zinc solubilization index (cm)	3.33	4.22
Potassium solubilization	−	−
ACC deaminase production	+	+
Nitrogen fixation	−	+
Hydrogen cyanide production	−	+
Biofilm production	+	+

Standard tests were performed to evaluate the isolates for different PGP traits and regular observations as per the assay protocols, with the most significant effects bolded. The values given are average measurements from three replications of each assay across three independent trials. A plus sign (+) indicates a positive test response, while a minus sign (−) denotes a negative response.

### ADJ1 and ADJ6 variously enhance wheat growth and productivity

While the results from the above growth promotion (PGP) assays indicate the potential capabilities of the two isolates, a thorough justification of these potentials is crucial for translational relevance. To meet the stated requirements, the effects of the ADJ1 and ADJ6 isolates on the growth and productivity of a commercially available wheat variety were thoroughly and variously investigated. The results of these various trials are presented below.

#### Improved wheat seed growth from in vitro priming with ADJ isolates' spent supernatants

**Figure 3:**
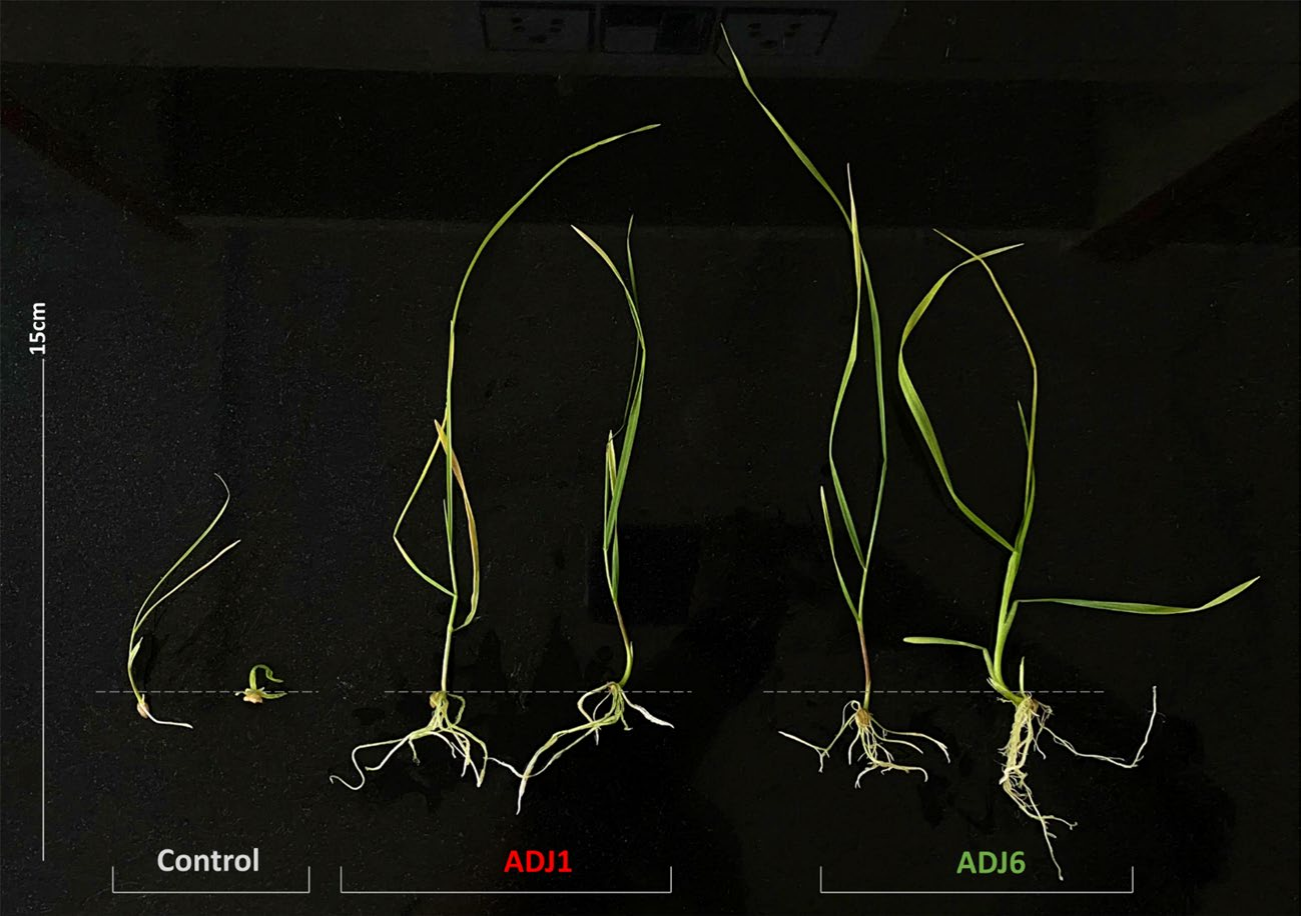
Two-week-old wheat seedlings grown from seeds primed in vitro with spent supernatants from either ADJ1 or ADJ6 alone. The endophyte-primed seeds germinated into seedlings with more developed shoots and roots compared to the unprimed control seedlings. This enhanced early growth indicates beneficial effects from seed priming with the ADJ isolates' spent supernatants.

In individual experiments, near aseptic wheat seeds were imbibed overnight in cell-free spent supernatants from NB-grown cultures of either ADJ1 and/or ADJ6. Control seed lots however were primed only with NB avoiding any bacterial inoculum. These primed seeds were then placed in PTC jam bottles containing MS media with culturing conditions like those used in PTC (see materials and methods). The use of clarified supernatants in priming significantly accelerated and improved germination responses in wheat seeds under these in vitro trials, as depicted in Fig. 3. Both ADJ1/ADJ6 priming exhibited an overall enhancement in seed germination, with primordial development seen within 3.0–3.5 days and approximately 80–95% successful germination. In contrast, control sets showed germination only by the 4th day, with a significantly lower extent (about 60–70% germination). So, this indicated that ADJ endophyte supernatants could enhance seed germination by the rate and number of germinating seedlings. Furthermore, subsequent effects from endophyte-spent supernatants could be witnessed more vividly 2 weeks post-in vitro seed establishment. Particularly, shoot and root length was seen to increase significantly incremented with secondary appendages as well compared to those in control seed sets. Roots showed drastic grith enhancement and were seen with more in numbers with a branching effect much earlier than the control would later exhibit. Our findings suggest that the plant growth-promoting benefits attributed to the priming treatments (with spent supernatants from either ADJ1 or ADJ6) likely transferred to crop plants. Notably, ADJ6 appeared more effective in promoting fair seedling growth in all trials (Fig. 3), as expected from its previously assessed plant growth promotion credentials (Table 1).

#### Seed priming facilitated by ADJ1 and ADJ6 leads to improved ex vitro growth in wheat

**Figure 4:**
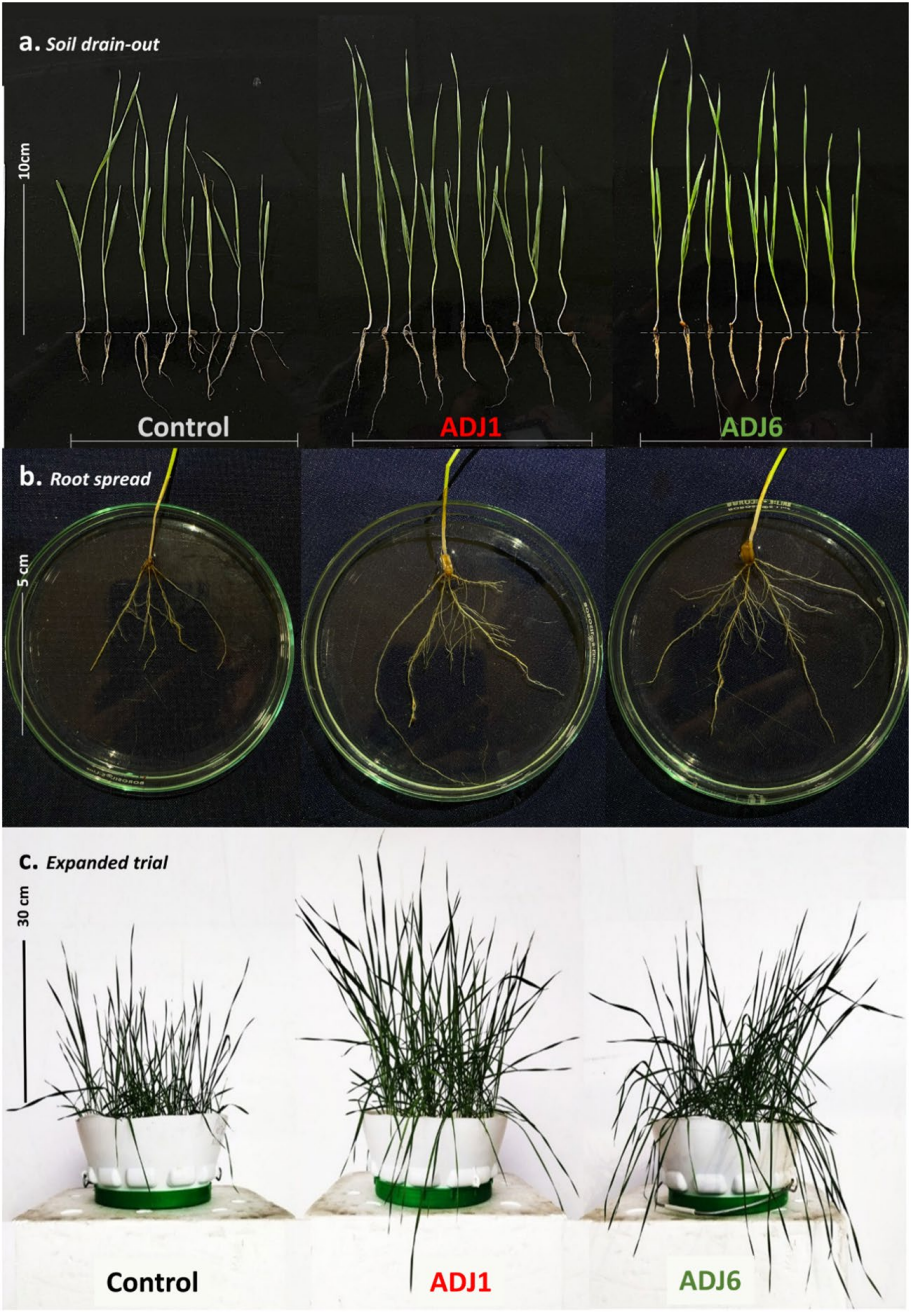
Investigating pre-treated wheat seeds with ADJ1/ADJ6 in ex vitro settings. In (**a**) seedling after soil drained out at 5 weeks of sowing; in (**b**) same [as shown in panel (**a**)] with roots spread apart over a Petri dish to show morphological features; and in (**c**), seedling growth at 8 weeks post sowing. Each document seedling response is shown as the mean representative of all the trials(s) respective to the experimental treatments. Scale bars on the left in each panel figure represent measured lengths for comparison in the same scope of view.

**Figure 5:**
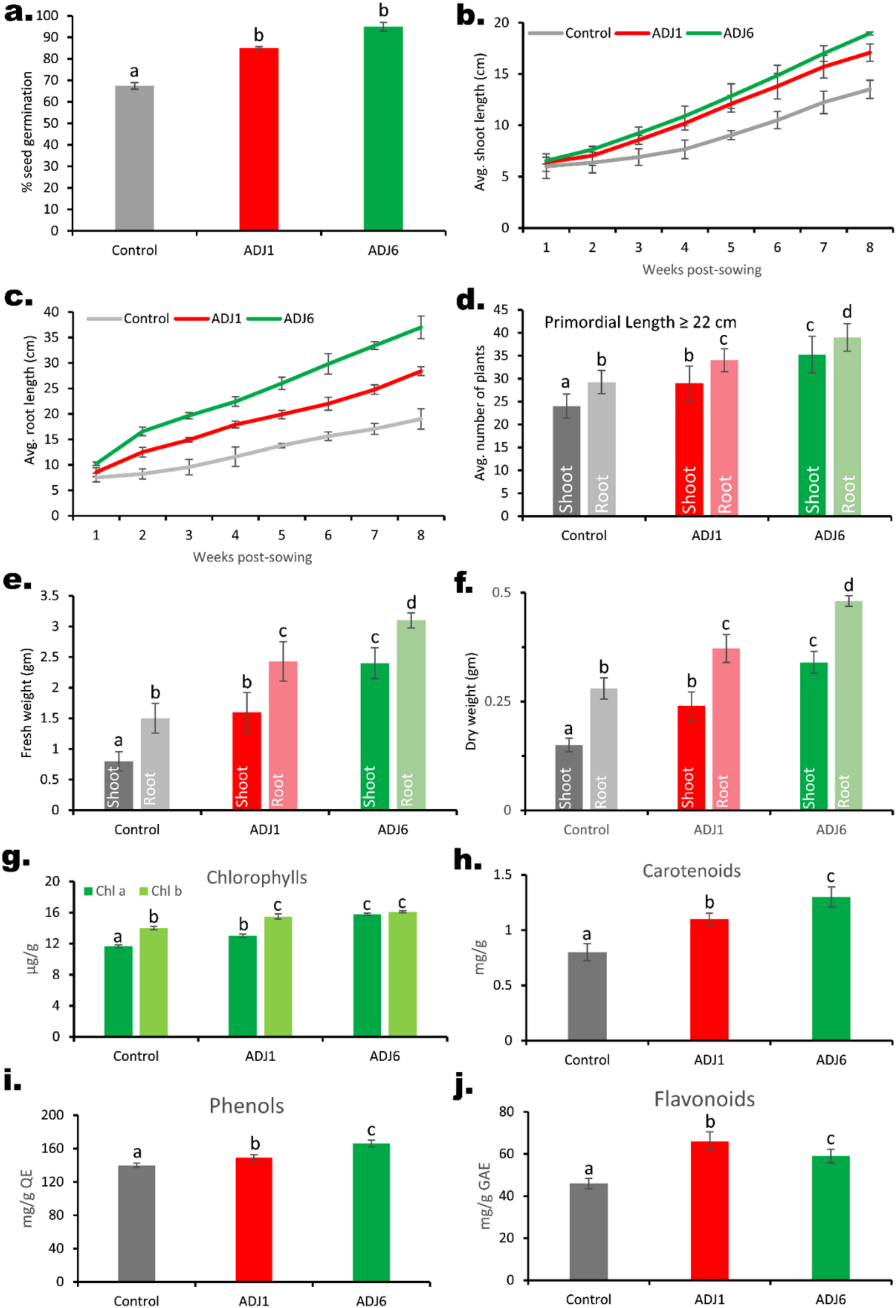
Drain-out assessments of wheat pre-treated with ADJ1/ADJ6 in ex vitro experiments. Continuing from figure herein (**a**), depicts seed germination profiles; (**b**,**c**), shoot and root lengths; (**d**) numbers of shoots and/or root primordia beyond a margin of 22 cm; (**e**,**f**) primordial fresh and dry weights, respectively; in (**g**–**j**) various physiological profiling parameters as shown with values of phenolic compounds expressed as equivalent to quercetin (QE) standard and similarly, those of flavonoids express equivalence to gallic acid (GAE). In each graph, the average values measured for each group are denoted with different superscript letters, indicating statistically significant differences between the groups as determined by Duncan's multiple range test at a 5% significance level (*p* < 0.05).

The outcomes observed from the in vitro evaluation of ADJ1/ADJ6 spent supernatant-primed wheat seeds prompted us to extend our investigation to assess whether the attributes of these PGPBs would also contribute to crop growth under ex vitro conditions. To commit to this, ex-vitro experiments were conducted employing a completely randomized design (CRD). Sets of 45 healthy wheat seeds each were separately treated with freshly grown overnight cultures of either ADJ1 or ADJ6, with the inoculum prepared in NB (as detailed earlier). Control seeds remained untreated with the endophytes. These treated seeds were sown in garden soil beds autoclaved and set over plastic tray pots. A weekly regimen was initiated, where plants in the ADJ1/ADJ6-bacterized treatments received an additional dose of freshly grown inoculum of the respective strains. Control trays, housing wheat seeds without bacterization treatment, were similarly moistened with comparable volumes of NB. This ex vitro tray experiment spanned 12 weeks, during which various growth parameters were recorded, namely, seed germination profiles, seedling morphometrics, and physiological profiles (Fig. 4, and Fig. 5).

**Figure 6:**
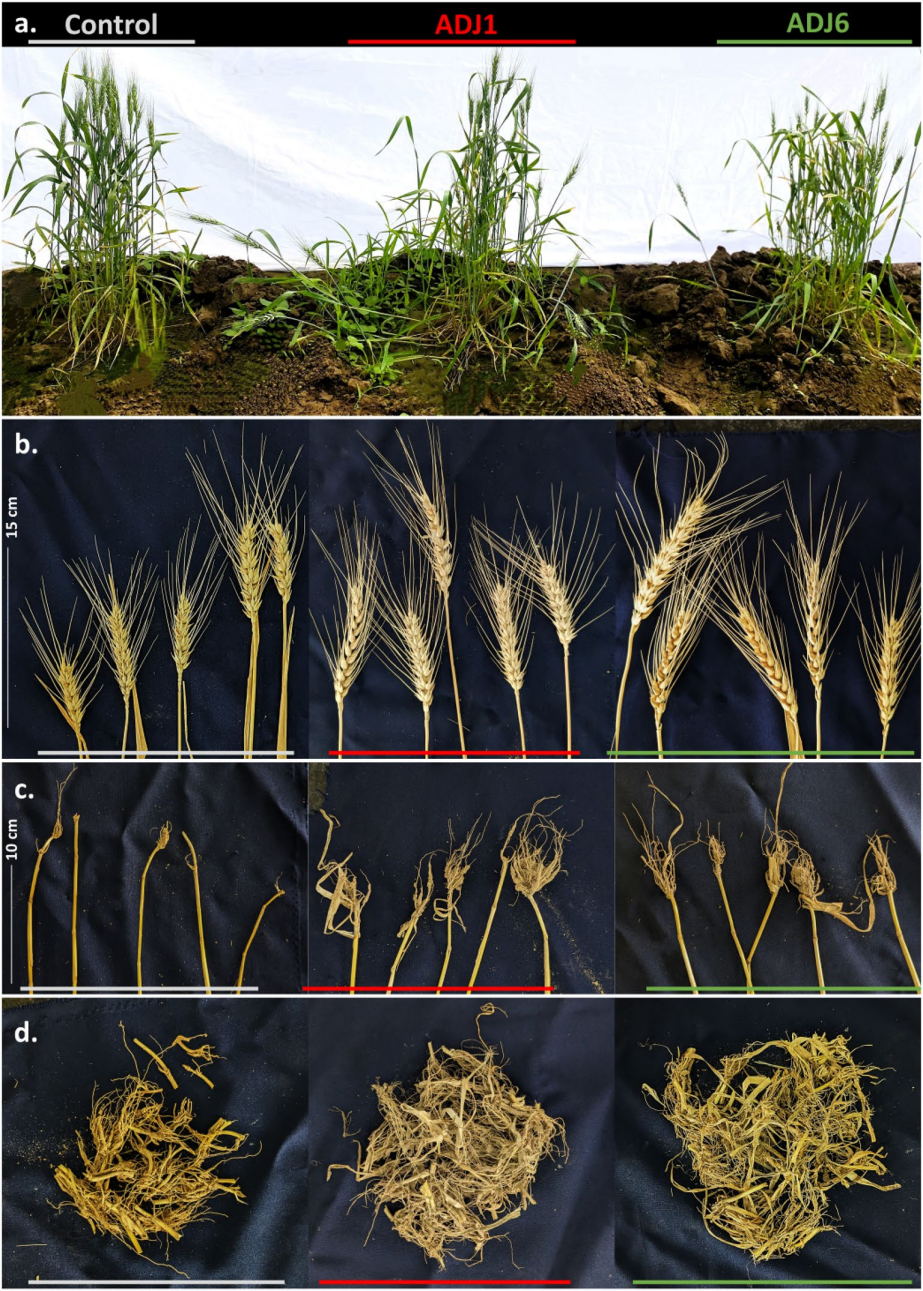
Field-grown wheat with ADJ1/ADJ6/control treatments. In (**a**) wheat at 14th-week post sowing with/without endophyte treatments; and rest of the panels shown after crop harvesting with differences in morphological traits amongst various experimental treatment differences as in (**b**) grain heads; in (**c**) pulled-out shoots showing the dexterity of the roots in endophyte treated wheat plants; in d, retrievable root biomass from the underneath of 5 pullet = out shoots from each trial set.

Consistent with the in vitro testing (Fig. 3), seed germination rates were notably increased in ADJ1/ADJ6-bacterized seed trays, with primordia visible within 2.5–3 days from sowing (Fig. 4 and Fig. 5). A higher germination extent was seen in both cases (ADJ1, approximately 85%; and ADJ6, approximately 95%) compared to the control treatments (approximately 65% germination, Fig. 5a) on the 3rd day post establishment. Early seedling emergence observed with bacterization suggests wholesomely enhanced plant growth, corroborated by the increase in the length of shoots over time (Fig. 5a–f,). Measurements taken 12 weeks after sowing showed that wheat growth was enhanced by both ADJ1 and ADJ6. At this stage, wheat seedlings treated with ADJ1/ADJ6 had significantly greater overall length, more shoots, more roots, more root branches, and thicker roots compared to untreated control seedlings. As shown in Figs. 4 and 5d–f, wheat grown with ADJ1/ADJ6 isolates exhibited increased growth metrics like shoot number, root number, root branching, and root width relative to the controls without bacterial treatment (Figs. 4, 5d–f). This provides evidence that the ADJ1 and ADJ6 isolates promoted growth when applied to wheat. Notably, the counts of shoots and root numbers surpassing a specified threshold (≥ 22 cm) were significantly higher in seedlings treated with bacterization-cum-booster dosing than in the control treatments (as shown in Fig. 6d). An interesting observation from the drain-out data was specifically related to root structure in all repeated tests. In control treatments, a substantial majority of roots (above 70–75%) were slender, with one main root displaying smaller branching along its length (Fig. 4b). In contrast, plantlets from ADJ1-bacterized-cum-booster-dosed treatments exhibited a higher branching extent along the length of the majority of main roots (about 80–85%), more pronounced at the tip. Wheat treated with ADJ6 showed particularly abundant rooting and root branching features in proximity to the shoot–root interface, as seen in Fig. 4b. The differences in root structure between the experimental groups were also reflected in the overall fresh and dry weight measurements from multiple trials, as shown in Fig. 5e,f. The wheat grown with ADJ6 had higher fresh and dry weights, further indicating increased root growth compared to controls.

To validate the augmented growth of wheat seedlings in ex vitro trials subjected to distinct treatments with either ADJ1 or ADJ6 (Fig. 4), various standard physiological parameters governing plant growth were scrutinized (Fig. 5g–j). The enhancements observed in the profile of photosynthetic pigments would inherently suffice to the overall physiological robustness of the crop^94^. Phenols are pivotal in shielding crops from a variety of abiotic stresses by antioxidation to knock down the effects of reactive oxygen species (ROS) and facilitate antioxidation^95^. Consequently, the total phenolic content exhibited a noteworthy increase in plants treated with ADJ1/ADJ6 except those in control treatments. Furthermore, the cumulative presence of total flavonoids encompasses the accumulation of specific secondary bioactive metabolites which mitigate diverse stressors^96^. The heightened physiological profiles observed under plant growth-promoting rhizobacteria (PGPR) treatments would positively impact the photosynthetic efficiency of plants, ultimately resulting in an upswing in overall plant productivity.

#### Field trials with wheat validate PGP prospects with ADJ1 and ADJ6

**Figure 7:**
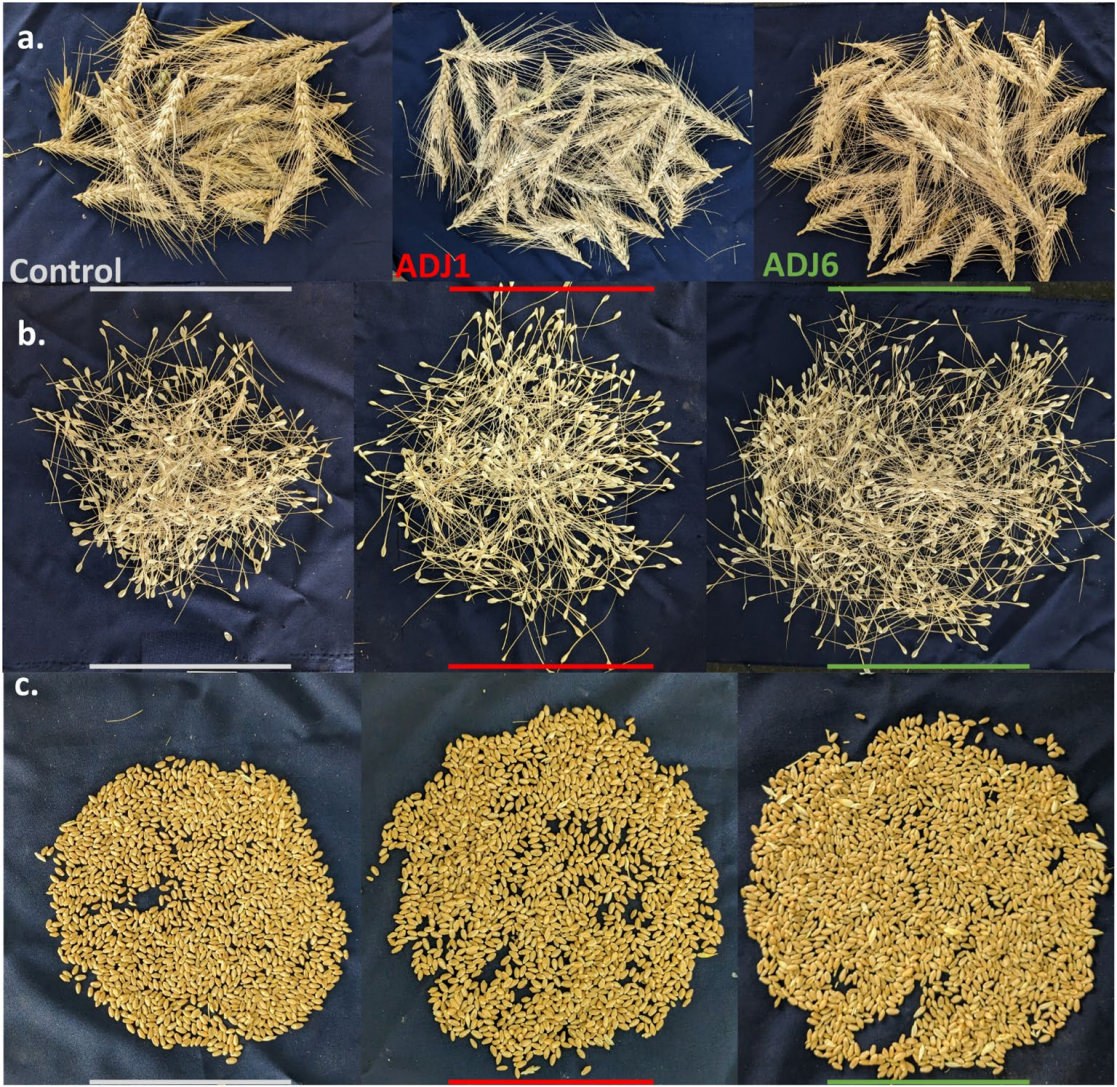
Productivity outcomes from field-trialed wheat. In (**a**) accumulated spikes (grain heads) from each trial; (**b**) sorted spikelets (grain heads) retrieved from the respective trial; and in (**c**) overall grain yield. Colored bars are used to extendedly mean different treatment groups in other panels as highlighted above (Control, ADJ1, and ADJ6).

In vitro and ex vitro trials involving seed treatments with ADJ1/ADJ6 demonstrated a significant improvement in both morphometric and physiological growth parameters in wheat (refer to Figs. 3, 4, 5). These results align with the previously assessed plant growth promotion (PGP) potentials (Table 1). Motivated by these findings, field trials were conducted on various crops treated with ADJ1 and ADJ6. Notably, successful field evaluations were recently completed on wheat, further validating the extended benefits in crop productivity. For field trials, ADJ1 and ADJ6-primed wheat seeds were established in soil beds in a polyhouse without any fertilizer or exogenous supplementation of other plant growth enhancers. Results are presented in Figs. 6, and 7 (and also in Supplementary Fig. 2). Like ex vitro trials, field-grown wheat crops from ADJ1/ADJ6 seed priming exhibited enhanced seed germination efficiency (Supplementary Fig. 2a). At the harvest time, overall shoot height above the ground was also seen comparatively higher in bacterized seed lots than in the controls (Supplementary Fig. 2b). Over the time in the field, shoot height increments mirrored trends seen in ex vitro trials, with significant differences recorded in most plants at 13 weeks post-sowing. During this instance, some propagules in the control group dried, indicating the need for harvest. In contrast, the ADJ1/ADJ6-treated sets, however, showed these observations only until a week later than the controls. After 16 weeks of growth, the wheat crops from all trial conditions were harvested and analyzed for morphological traits and yield. In brief, ADJ1/ADJ6 priming treatments resulted in more pronounced size increments in the spikes, stalk, and root regions of the plants post-harvest (Figs. 6, 7 and in Supplementary Fig. 2). Examination of root sections inferred a longer rooting feature in ADJ1/ADJ6-treated plants compared to control-treated plants, despite potential damage during pull-outs (Fig. 6c,d, and in Supplementary Fig. 2c). This suggests that ADJ1/ADJ6 priming contributed to better rooting, maintaining greater strength during pull-out maneuvers than control-treated plants. Differences in average primordial dry weights further supported this observation (Supplementary Fig. 2e,f). The spikes were slightly more sizeable (Fig. 6b, and Supplementary Fig. 2d) showing overall incremented length under ADJ1/ADJ6 treatments compared to those of the control treatment. From sowing to crop harvest across the 4-month trials, the percentage of wheat crop survival was higher in a steady manner. Importantly, these effects, along with the PGP benefits, sufficed to overall more significantly improve plant productive growth (Fig. 7a–c, and in Supplementary Fig. 2g,h). It would be interesting to further assess the crop quality in terms of nutritional attributes as well as inherent bioactives’ differential profile following endophytes’ treatments.

#### ADJ endophytes could grow under high-saline medium

**Figure 8:**
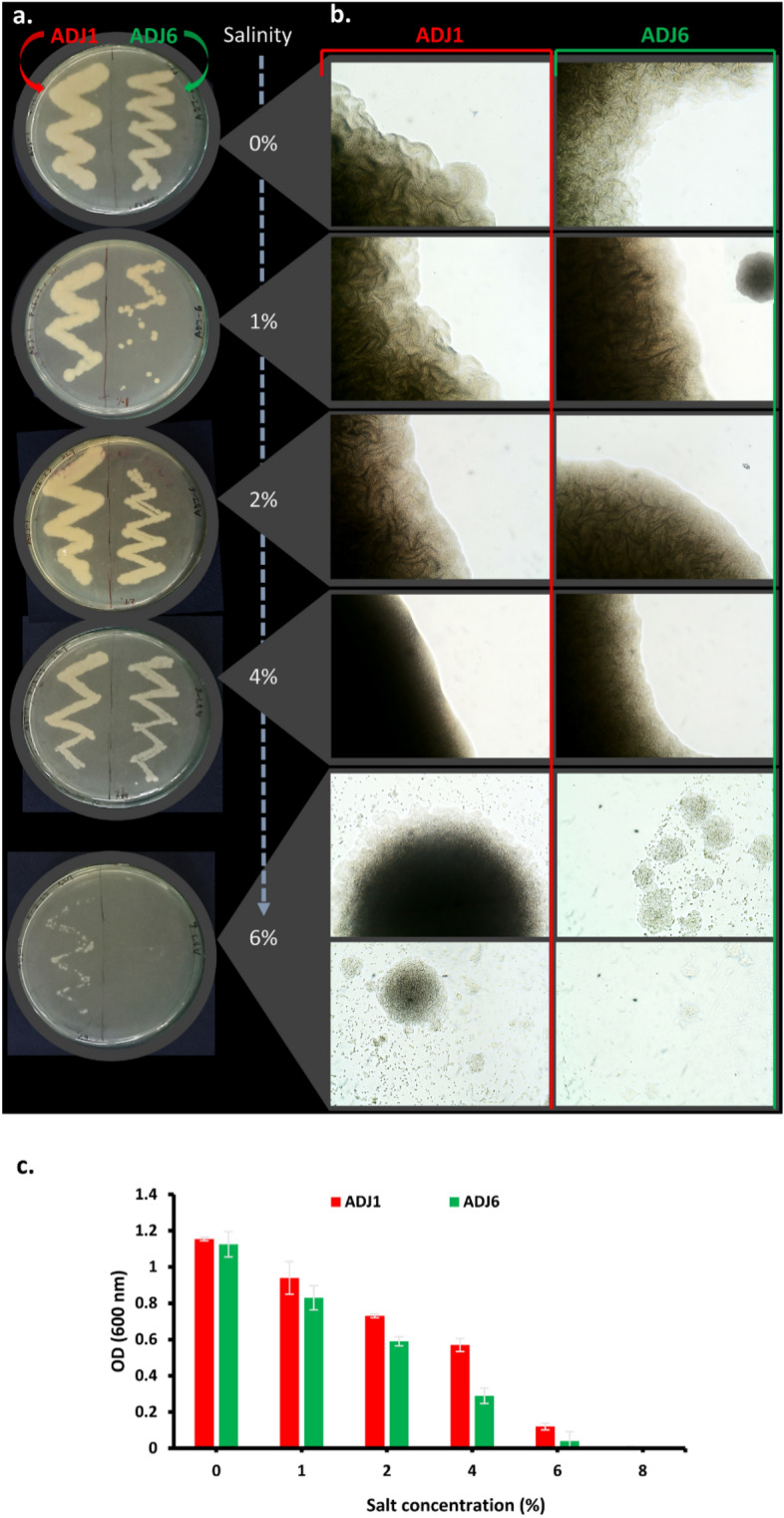
Salinity tolerance profile of ADJ endophytes. In (**a**) ADJ1 and ADJ6 streak plated over NA, in (**b**) respectively, a close microscopic view (100× magnification) of their swarming growth over the streak or growth retardation under increasing salinity in culture media; and in (**c**) similarly, profile produced over NB with various salt concentrations measured with spectrophotometry (OD_600nm_). All observations developed post overnight incubations of inoculations in nutrient media at 37 °C, dark (in (**c**) with orbital shaking at 150 rpm for 18 h). All experiments were repeated at least thrice and values depicting the mean of three or more trials with standard deviation were shown as error bars. Also in panel (**c**) the average values measured for each group are denoted with different superscript letters, indicating statistically significant differences between the groups as determined by Duncan's multiple range test at a 5% significance level (*p* < 0.05).

The two ADJ endophytes, ADJ1 and ADJ6, demonstrated the capability to grow across a range of salt concentrations (1–8%) in both liquid and agar-supplemented semisolid bacteriological media (Fig. 8). On streak-plated culture inoculums (Fig. 8a), both endophytes exhibited nearly identical streaks at 1% and 2% salt without subtle deformations compared to the control (0% supplementation; NA, however, was canonically made with 0.5% NaCl), indicating fair growth for both endophytes. Progressing to 4% supplemented salinity in media (Fig. 8a), streaks showed reduced haziness, signaling reduced but viable growth at this salt concentration. ADJ1's streak appeared thicker than that of ADJ6. At 6% salinity, the two endophytes exhibited distinguishable salinity tolerance capacities; ADJ1 displayed restricted streak growth with intermittent development of colonies, while ADJ6's growth was significantly more affected. Beyond 8% salt, both endophytes ceased to show any growth, indicating susceptibility at this salinity regime (data not shown). Figure 8b illustrates the microscopic view (at 10× magnification) of the endophytes' growth at various salt concentrations on the plate. The figures vividly show the loss of swarming features at 6% salinity for ADJ6 but not for ADJ1, which seemed to adapt somehow to the high salt in the media. This observation is more clearly documented in liquid culture treatments with salt for both endophytes (Fig. 8c). Overnight incubation revealed fair growth up to 4% (= 684.44 mM) NaCl in the media for both isolates, but beyond this concentration, the conditions for ADJ6 seemed severely limiting for growth to reach an appreciable optical density (OD).

#### ADJ endophytes facilitated wheat seedling growth under high salinity

**Figure 9:**
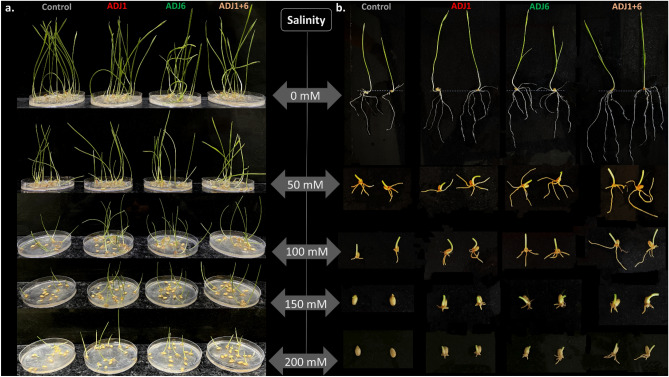
In vitro rescue of wheat seedling growth under salinity stress by ADJ endophytes. In a, Shoot growth profiles and in b, root growth profiles of wheat seeds germination trial under the effect of various concentrations of salt in the medium and the growth enhancement attribute from the PGPBs exclusively (ADJ1 and ADJ6) and as a consortium (ADJ1 + 6). All observations were recorded at 3 weeks post germination setting and each image panel represents the mean effect. Note the PGP effect of the two endophytes in exclusivity and their combinatorial treatment with seeds seen in enhanced morphometrics in shoot and root maintained with and without salinity stress. Note that in panel (**b**) shoot segments were trimmed off from the seed to accommodate the panel frames and focus the root primordial growth.

This study explores the in vitro priming effects of novel isolates, ADJ1 and ADJ6, along with their consortium (ADJ1 + 6), on wheat seedlings under varying salinity conditions (50 mM, 100 mM, 150 mM, and 200 mM) (Fig. 9 and Supplementary Fig. 3). While the bacterial endophytes demonstrated moderate salt tolerance (Fig. 8), their impact on wheat seedling growth, particularly in shoots, was evaluated in comparison to untreated control seed lots (Fig. 9a). As expected in control treatments (wheat seeds untreated with either bacteria or the consortium preparation), under zero salinity in the germination medium, seed germination extent (Supplementary Fig. 3a) and the shoot and root growth enhancements (Fig. 9a,b and Supplementary Fig. 3b) are less pronounced compared to those with the three seed bacterized test sets. This was expected from endophytes’ PGP potentials previously assayed (Table 1) and variously tested with seed priming treatments under in vitro, ex vitro, and field experiments (Figs. 3, 4, 5, 6, 7, 8, 9). Under germination media salinity profiles, as seen with trials outlined here (Fig. 9 and Supplementary Fig. 3), it is understandable that ADJ1, ADJ6, and the consortium exerted a positive influence on seed germination, even under the lowest salinity level of 50 mM. Enhanced germination rates were particularly pronounced with either the two, ADJ1 and ADJ6 in separate treatments or their consortium, demonstrating their potential in promoting early developmental stages. Morphometric analyses revealed notable enhancements in shoot and root lengths (Fig. 9 and Supplementary Fig. 3a,b), particularly with ADJ6 and the consortium, suggesting potential plant growth-promoting (PGP) effects. As salinity levels escalated, the deleterious effects on seedling growth became apparent (Fig. 9). However, seed priming with ADJ1 and ADJ6 mitigated these effects, with ADJ6 showcasing a more substantial impact. Shoot growth, while reduced under salinity stress, exhibited a more gradual decline compared to root growth, suggesting a differential response to salinity in wheat seedlings. ADJ6 was seen to enhance the rate of shoot development more efficiently than ADJ1 as surmised from the observed two-leaf primordial developments (Fig. 9b).

Although shoot growth at various salinity profiles of the germination media was productively maintained, root growth was gravely impacted throughout the trials (Fig. 9). Although root growth was not very productive even in the case of Adj1/ADJ6 bacterization over wheat seeds, remarkably significant rescue could be accorded to the consortium-(ADJ1 + 6)-assisted treatments (Fig. 9a, and Supplementary Fig. 3b). The latter synergistic PGP effect of the consortium on the wheat seedling roots can be easily witnessed at the 50- and 100-mM salinity regime, probably this partly rescues the plant stature under stress. This prospect could be useful for application in crop biotechnology, as it is known that at 50–100 mM salinity in soil substratum (5–10 dsm^−1^), wheat productivity is deleteriously reduced. Elevated salinity levels postpone the initiation of seedling germination, diminish both the growth of seedlings and the spread of germination occurrences, as well as influence seedling metabolism^97–100^. Various physiological parameters, viz., total chlorophyll, carotenoids, phenols, flavonoids, and proline, assessed in our salinity stress trials over bacterized wheat seeds show that at every higher salt concentration, detriment from salinity stress was kept under profound check comparatively to that seen under control treated wheat seeds (Supplementary Fig. 3c–i). The above results with wheat plants and the notion of bacterial tolerance to moderate salt (up to 4%) have promiscuously intrigued several field-based trials using salt regimes in experimental soils and those from various regions with high salinity portfolios. These trials are further being carried out at our end.

## Discussion

The urgent need to ensure global food security in the face of climate change is driving increased scientific interest in exploring microorganisms from stress-resilient plants for their bioprospecting potential^101–105^. Our research theme explores bioprospecting avenues with many neglected and underutilized plants, their stress tolerance regulation, and their intrinsic microbial partnerships, with the overarching goal of deciphering and applying their biopotentials to enhance sustainable, environmentally friendly, and economically viable agricultural crop productivity and their valorization with translational outcomes^31,35,65,106–109^. Agave plants offer an intriguing avenue for studying the resilient succulent plant lifestyle and their possession of microbial assets for translational bioprospecting. Despite considerable research on other aspects, information on endophyte communities associated with the species *A. desmettiana* has been absent in the literature. The presented study addresses this gap by documenting the first ever reported isolation and identification of plant growth-promoting microbial endophytes, namely ADJ1 and ADJ6, corresponding to *B. cereus* and *P. aryabhattai*, respectively. *Priestia* species were part of the *Bacillus* genus before^110^, but later taxonomic rearrangements had redefined the latter genus, which now includes only closely related *B. subtilis* and *B. cereus*^111,112^. Genus *Priestia* distinctly contains 10 identified species as of May 2021 (https://lpsn.dsmz.de/genus/priestia) which can grow in temperatures ranging from 5 to 48 °C, thus adaptng them to diverse habitats. Of these, *P. aryabhattai* is known for its potential bioremediation applications^113,114^. It is quite interesting that although *Priestia* and *Bacillus* are taxonomically distinct genera, the ADJ1 and ADJ6 isolates staged in this study, were recovered from the agave leaf as a mixed culture. Our work transitioned from lab studies to field testing of wheat seed treatments with the endophytes ADJ1 and ADJ6. The study revealed their promising prospects into enhancing crop productivity. reatments with ADJ6 comparatively showed more enhanced wheat growth promotion and productivity parameters than those with ADJ1. As with the ADJ1 and AdJ6 isolates, a substantial number of other *B. cereus*^115–121^ and *P. aryabhattai*^122–127^ have been shown with PGP assets.

Agaves generally display varying degrees of tolerance to salt, with the extent differing among different species and varieties^128,129^. Also, many agave species naturally thrive in arid regions where they frequently encounter soils with relatively high salinity levels. These plants have evolved specific mechanisms enabling inherent osmotic adjustments and/or salt exclusions^130,131^. Given that many endophytes mimic host plant adaptations to mitigate stress^132,133^ and can assist the host in stress management, it became intriguing to explore whether the isolated agave endophytes (ADJ1 and ADJ6) exhibit tolerance to high saline environments and, if so, whether this attribute facilitates the rescue of plants under subtle salinity stress. Our study observed promising growth of ADJ1 and ADJ6 across varying salt concentrations in both liquid and agar-supplemented media. Notably, both isolates exhibited commendable growth at lower salt concentrations (1–2%), showcasing their adaptability to moderately saline environments. As the salinity levels increased (4–6%), ADJ1 demonstrated a relatively sustained growth, while ADJ6 exhibited a more pronounced sensitivity. The microscopic analysis further resolved these differences, into showing ADJ6 losing its swarming features at 6% salinity, contrasted well with ADJ1’s adaptability. Further. the in vitro priming effects of ADJ1 and ADJ6 were assessed individually and in consortium (ADJ1 + 6), on wheat seedlings, with a particular emphasis on shoot growth under various salinity levels. The observed enhancements in seed germination and seedling growth, particularly in shoots, imply a potential PGP effect conferred by the isolates. The greater efficacy of ADJ6, and that even more with the consortium (ADJ1 + 6) suggests possible synergistic effects among the bacterial strains, warranting further investigation into their cooperative actions. Both ADJ1 and ADJ6 show PGP traits, including the production of phytohormones, nitrogen fixation, and phosphate solubilization. These mechanisms likely contribute to the observed improvements in germination and seedling growth. ADJ6 may have a more pronounced impact due to its specific PGP capabilities. The superior performance of the consortium (ADJ1 + 6) suggests potential synergies among ADJ1 and ADJ6. The cooperative actions of these endophytes may contribute to a more robust response to salinity stress, emphasizing the importance of exploring microbial consortia for agricultural applications. At the outset, the in vitro priming effects observed in this study signify a promising avenue for harnessing the PGP potential of ADJ1 and ADJ6 to enhance wheat crop resilience to salinity stress. Further research, including comprehensive field trials, molecular analyses, and safety assessments, will be crucial for advancing these findings from the laboratory to practical and sustainable agricultural applications.

## Conclusions

Agave species have garnered significant attention in stress biology due to their remarkable resilience across diverse environmental conditions. This inherent resilience positions them as critical plant candidates for translational research endeavors, spanning phytomedicines, bioenergy production, phytoremediation, and agribiotechnology. While notable progress has been made in understanding microbial associations with various agave species, a substantial knowledge gap persists in the literature regarding endophyte communities specifically related to *A. desmettiana*. This pioneering study effectively bridges this gap by unveiling, for the first time, growth-promoting microbial endophytes associated with this species. Our findings highlight promising prospects for enhancing crop productivity through seed-priming treatments with these novel microbial endophytes. Notably, the ADJ6 isolate demonstrated more pronounced effects compared to control treatments, indicating its potential as a potent bioinoculant. Synergistically, the two isolates exhibited the ability to mitigate salinity stress in wheat, underscoring their potential for developing sustainable agricultural practices. In a broader context, it is crucial to prioritize and invest in comprehensive research initiatives to fully comprehend and harness the biotechnological potential of agave plants, ensuring their contributions to a resilient and sustainable future. These translational research endeavors hold particular importance in light of the global objective to achieve no poverty (SDG 1), zero hunger (SDG 2), responsible consumption and production (SDG 12) and climate action (SDG 13) by 2030, amidst impending challenges associated with meeting the broader Sustainable Development Goals (SDGs) delineated by the United Nations.

### Supplementary Information

Supplementary Information.

## Data Availability

Homologous sequences of the 16S rRNA gene for ADJ1 and ADJ6 isolates have been submitted to NCBI's GenBank under accession numbers OR898198 and OR898199, respectively. The corresponding authors are committed to providing any raw data supporting the findings of this article upon formal request, without any undue reservation.

## References

[1] Ren K (2023). Achieving high yield and nitrogen agronomic efficiency by coupling wheat varieties with soil fertility. Sci. Total Environ..

[2] He Y (2024). Climate change enhances stability of wheat-flowering-date. Sci. Total Environ..

[3] Guo X, Zhang P, Yue Y (2024). Prediction of global wheat cultivation distribution under climate change and socioeconomic development. Sci. Total Environ..

[4] Kheir AMS (2019). Impacts of rising temperature, carbon dioxide concentration and sea level on wheat production in North Nile delta. Sci. Total Environ..

[5] van den Burg S (2024). Knowledge gaps on how to adapt crop production under changing saline circumstances in the Netherlands. Sci. Total Environ..

[6] Ibarra-Villarreal AL (2021). Salt-tolerant Bacillus species as a promising strategy to mitigate the salinity stress in wheat (*Triticum turgidum* subsp. durum). J. Arid Environ..

[7] Cheng Z, Chen Y, Zhang F (2018). Effect of reclamation of abandoned salinized farmland on soil bacterial communities in arid northwest China. Sci. Total Environ..

[8] Duan M (2023). Integrated microbiological and metabolomics analyses to understand the mechanism that allows modified biochar to affect the alkalinity of saline soil and winter wheat growth. Sci. Total Environ..

[9] Shafi M, Khan MJ, Bakht J, Khan MA (2013). Response of wheat genotypes to salinity under field environment. Pak. J. Bot..

[10] Ondrasek G, Rengel Z (2021). Environmental salinization processes: Detection, implications & solutions. Sci. Total Environ..

[11] Lago-Olveira S, Rebolledo-Leiva R, Garofalo P, Moreira MT, González-García S (2023). Environmental and economic benefits of wheat and chickpea crop rotation in the Mediterranean region of Apulia (Italy). Sci. Total Environ..

[12] Li M (2024). Balancing grain yield and environmental performance by optimizing planting patterns of rice–wheat cropping systems. Sci. Total Environ..

[13] Saeed T (2023). Exploring the effects of selenium and brassinosteroids on photosynthesis and protein expression patterns in tomato plants under low temperatures. Plants.

[14] Debnath S (2023). The enhanced affinity of WRKY reinforces drought tolerance in *Solanum lycopersicum* L.: An innovative bioinformatics study. Plants.

[15] Verma S, Negi NP, Pareek S, Mudgal G, Kumar D (2022). Auxin response factors in plant adaptation to drought and salinity stress. Physiol. Plant..

[16] Naik K, Mishra S, Srichandan H, Singh PK, Sarangi PK (2019). Plant growth promoting microbes: Potential link to sustainable agriculture and environment. Biocatal. Agric. Biotechnol..

[17] Alves ARA, Yin Q, Oliveira RS, Silva EF, Novo LAB (2022). Plant growth-promoting bacteria in phytoremediation of metal-polluted soils: Current knowledge and future directions. Sci. Total Environ..

[18] Ruzzi M, Aroca R (2015). Plant growth-promoting rhizobacteria act as biostimulants in horticulture. Sci. Hortic..

[19] Pathania P, Rajta A, Singh PC, Bhatia R (2020). Role of plant growth-promoting bacteria in sustainable agriculture. Biocatal. Agric. Biotechnol..

[20] Singh GB (2021). Plant-Microbial Interactions and Smart Agricultural Biotechnology.

[21] Anand U (2023). Current scenario and future prospects of endophytic microbes: Promising candidates for abiotic and biotic stress management for agricultural and environmental sustainability. Microbial. Ecol..

[22] Afridi MS (2019). Induction of tolerance to salinity in wheat genotypes by plant growth promoting endophytes: Involvement of ACC deaminase and antioxidant enzymes. Plant Physiol. Biochem..

[23] Prajapati P, Yadav M, Nishad JH, Gautam VS, Kharwar RN (2024). Salt tolerant fungal endophytes alleviate the growth and yield of saline-affected wheat genotype PBW-343. Microbiol. Res..

[24] Nagrale DT (2023). PGPR: The treasure of multifarious beneficial microorganisms for nutrient mobilization, pest biocontrol and plant growth promotion in field crops. World J. Microbiol. Biotechnol..

[25] Yadav J, Srivastva AK, Singh R (2024). Diversity of halotolerant endophytes from wheat (*Triticum aestivum*) and their response to mitigate salt stress in plants. Biocatal. Agric. Biotechnol..

[26] Díaz Herrera S, Grossi C, Zawoznik M, Groppa MD (2016). Wheat seeds harbour bacterial endophytes with potential as plant growth promoters and biocontrol agents of Fusarium graminearum. Microbiol. Res..

[27] Mishra P, Mishra J, Arora NK (2021). Plant growth promoting bacteria for combating salinity stress in plants—Recent developments and prospects: A review. Microbiol. Res..

[28] Faskhutdinova E (2024). Extremophilic bacteria as biofertilizer for agricultural wheat. J. Foods Raw Mater..

[29] Ringelberg D, Foley K, Reynolds CM (2012). Bacterial endophyte communities of two wheatgrass varieties following propagation in different growing media. Can. J. Microbiol..

[30] Mudgal G, Kaur J, Chand K, Singh GB, Kesari KK, Jha NK (2021). The antioxidant arsenal against COVID-19. Free Radical Biology and Environmental Toxicity.

[31] Parashar M (2023). Two novel plant-growth-promoting *Lelliottia amnigena* isolates from *Euphorbia prostrata* aiton enhance the overall productivity of wheat and tomato. Plants.

[32] Antunes GDR (2019). Associative diazotrophic bacteria from forage grasses in the Brazilian semi-arid region are effective plant growth promoters. J. Crop Pasture Sci..

[33] Eke P (2019). Endophytic bacteria of desert cactus (*Euphorbia trigonas* Mill) confer drought tolerance and induce growth promotion in tomato (*Solanum lycopersicum* L.). Microbiol. Res..

[34] Zhang Q, White JF (2021). Bioprospecting desert plants for endophytic and biostimulant microbes: A strategy for enhancing agricultural production in a Hotter, Drier Future. Biology.

[35] Kaur J (2022). An exopolysaccharide-producing novel *Agrobacterium pusense strain* JAS1 isolated from snake plant enhances plant growth and soil water retention. Sci. Rep..

[36] Mahgoub HAM, Fouda A, Eid AM, Ewais EE-D, Hassan SE-D (2021). Biotechnological application of plant growth-promoting endophytic bacteria isolated from halophytic plants to ameliorate salinity tolerance of *Vicia faba* L. Plant Biotechnol. Rep..

[37] Li X (2016). The endophytic bacteria isolated from elephant grass (*Pennisetum purpureum* Schumach) promote plant growth and enhance salt tolerance of Hybrid Pennisetum. Biotechnol. Biofuels.

[38] Bergsten SJ, Koeser AK, Stewart JR (2016). Evaluation of the impacts of salinity on biomass and nutrient levels of Agave species with agricultural potential in semiarid regions. HortScience.

[39] Raya FT (2021). Extreme physiology: Biomass and transcriptional profiling of three abandoned Agave cultivars. Ind. Crops Products.

[40] Nobel PS (1990). Environmental influences on CO_2_ uptake by agaves, cam plants with high productivities. Econ. Bot..

[41] Nabhan GP (2020). An Aridamerican model for agriculture in a hotter, water scarce world. Plants People Planet.

[42] Davis SC (2022). *Agave americana*: Characteristics and potential breeding priorities. Plants.

[43] Davis SC, Ortiz-Cano HG (2023). Lessons from the history of Agave: Ecological and cultural context for valuation of CAM. Ann. Bot..

[44] LaFevor MC (2014). Restoration of degraded agricultural terraces: Rebuilding landscape structure and process. J. Environ. Manag..

[45] Negi VS (2022). Land restoration in the Himalayan region: Steps towards biosphere integrity. Land Use Policy.

[46] Mendoza-Hernández PE, Orozco-Segovia A, Meave JA, Valverde T, Martínez-Ramos M (2013). Vegetation recovery and plant facilitation in a human-disturbed lava field in a megacity: Searching tools for ecosystem restoration. Plant Ecol..

[47] Arias-Medellín LA, Bonfil C, Valverde T (2016). Demographic analysis of *Agave angustifolia* (Agavaceae) with an emphasis on ecological restoration. Bot. Sci..

[48] Stewart JR (2015). Agave as a model CAM crop system for a warming and drying world. Front. Plant Sci..

[49] Le Houerou HN (2000). Utilization of fodder trees and shrubs in the arid and Semiarid zones of West Asia and North Africa. Arid Soil Res. Rehabilit..

[50] Ramana S (2022). Phytoremediation of soils contaminated with cadmium by *Agave americana*. J. Natl. Fibers.

[51] Machado-Estrada B, Calderón J, Moreno-Sánchez R, Rodríguez-Zavala JS (2013). Accumulation of arsenic, lead, copper, and zinc, and synthesis of phytochelatins by indigenous plants of a mining impacted area. Environ. Sci. Pollut. Res..

[52] Dhar S, Kaur J, Mudgal G (2021). Unveiling the multifaceted exploration from genomic insights to functional applications of the Agave genus: A comprehensive review. Natl. Volatiles Essent. Oils.

[53] Marone MP, Campanari MFZ, Raya FT, Pereira GAG, Carazzolle MF (2022). Fungal communities represent the majority of root-specific transcripts in the transcriptomes of Agave plants grown in semiarid regions. PeerJ.

[54] Beltran-Garcia MJ (2014). Nitrogen acquisition in *Agave tequilana* from degradation of endophytic bacteria. Sci. Rep..

[55] De Souza JT (2021). Endophytic bacteria isolated from both healthy and diseased *Agave sisalana* plants are able to control the bole rot disease. Biol. Control.

[56] Martinez-Rodriguez, A. *et al.* in *Seed Endophytes: Biology and Biotechnology* (Eds. Verma, S. K. & White Jr, J. F.) pp. 139–170 (Springer, 2019). 10.1007/978-3-030-10504-4_8.

[57] Coleman-Derr D (2016). Plant compartment and biogeography affect microbiome composition in cultivated and native Agave species. New Phytol..

[58] Damasceno CL (2019). Postharvest biocontrol of anthracnose in bananas by endophytic and soil rhizosphere bacteria associated with sisal (*Agave sisalana*) in Brazil. Biol. Control.

[59] Desgarennes D, Garrido E, Torres-Gomez MJ, Peña-Cabriales JJ, Partida-Martinez LP (2014). Diazotrophic potential among bacterial communities associated with wild and cultivated Agave species. FEMS Microbiol. Ecol..

[60] Obledo EN (2003). Increased photosyntethic efficiency generated by fungal symbiosis in Agave victoria-reginae. Plant Cell Tissue Organ Culture.

[61] Singh M, Srivastava M, Kumar A, Singh AK, Pandey KD, Kumar A, Singh VK (2020). Endophytic bacteria in plant disease management. Microbial Endophytes.

[62] Gibernau M, Chouteau M, Lavallée K, Barabé D (2010). Notes on the phenology, morphometry and floral biology of *Anaphyllopsis americana*. J. Int. Aroid Soc..

[63] Osborne, J. F. & Singh, D. Sisal and other long fiber agaves. in *Hybridization of Crop Plants*, pp. 565–575 (1980). 10.2135/1980.hybridizationofcrops.c40.

[64] Mansour H, Abou Dahab T, Ahmed A (2007). Studies on some cacti and succulents, and their use in Egyptian botanic gardens. 1. Effect of salinity levels and fertilization on vegetative growth and leaf anatomical structure of Agave Sisalana Perrine plants. J. Product. Dev..

[65] Kaur J, Mudgal G (2021). An efficient and quick protocol for in vitro multiplication of snake plant, *Sansevieria trifasciata* var. Laurentii [Prain]. Plant Cell Tissue Organ Culture.

[66] Sahoo B, Ningthoujam R, Chaudhuri S (2019). Isolation and characterization of a lindane degrading bacteria *Paracoccus* sp. NITDBR1 and evaluation of its plant growth promoting traits. Int. Microbiol..

[67] Cappuccino, J. & Sherman, N. Biochemical activities of microorganisms. *Microbiology, A Laboratory Manual. The Benjamin/Cummings Publishing Co. California, USA* 188–247 (1992).

[68] Bergey DH (1994). Bergey’s Manual of Determinative Bacteriology.

[69] Kang KH, Kim JK (2015). Degradation characteristics of a novel multi-enzyme-possessing *Bacillus licheniformis* TK3-Y strain for the treatment of high-salinity fish wastes and green seaweeds. Aquat. Sci..

[70] Ghasemi Y (2011). Screening and isolation of extracellular protease producing bacteria from the Maharloo Salt Lake. Iran. J. Pharm. Sci..

[71] Bharadwaj PS, Udupa PM (2019). Isolation, purification and characterization of pectinase enzyme from *Streptomyces thermocarboxydus*. J. Clin. Microbiol. Biochem. Technol..

[72] Abd-Elhalem BT, El-Sawy M, Gamal RF, Abou-Taleb KA (2015). Production of amylases from *Bacillus amyloliquefaciens* under submerged fermentation using some agro-industrial by-products. Ann. Agric. Sci..

[73] UK, G. Open consultation UK SMI ID 06: open consultation draft.

[74] Hudzicki J (2009). Kirby–Bauer disk diffusion susceptibility test protocol. Am. Soc. Microbiol..

[75] Humphries R, Bobenchik AM, Hindler JA, Schuetz AN (2021). Overview of changes to the clinical and laboratory standards institute performance standards for antimicrobial susceptibility testing, M100. J. Clin. Microbiol..

[76] Tamura K, Stecher G, Kumar S (2021). MEGA11: Molecular evolutionary genetics analysis version 11. Mol. Biol. Evolut..

[77] Muthuraja R, Muthukumar T (2021). Isolation and characterization of potassium solubilizing *Aspergillus* species isolated from saxum habitats and their effect on maize growth in different soil types. Geomicrobiol. J..

[78] Nautiyal CS (1999). An efficient microbiological growth medium for screening phosphate solubilizing microorganisms. FEMS Microbiol. Lett..

[79] Dworkin M, Foster J (1958). Experiments with some microorganisms which utilize ethane and hydrogen. J. Bacteriol..

[80] Cabaj A, Kosakowska A (2009). Iron-dependent growth of and siderophore production by two heterotrophic bacteria isolated from brackish water of the southern Baltic Sea. Microbiol. Res..

[81] Qing-Ping H, Jian-Guo X (2011). A simple double-layered chrome azurol S agar (SD-CASA) plate assay to optimize the production of siderophores by a potential biocontrol agent *Bacillus*. Afr. J. Microbiol. Res..

[82] Bent E, Tuzun S, Chanway CP, Enebak S (2001). Alterations in plant growth and in root hormone levels of lodgepole pines inoculated with rhizobacteria. Can. J. Microbiol..

[83] Gordon SA, Weber RP (1951). Colorimetric estimation of indoleacetic acid. Plant Physiol..

[84] Saravanan, V., Kumar, M. R. & Sa, T. Microbial zinc solubilization and their role on plants. In *Bacteria in Agrobiology: Plant Nutrient Management* 47–63. 10.1007/978-3-642-21061-7_3 (2011).

[85] Pandey PK, Samanta R, Yadav RNS (2015). Plant beneficial endophytic bacteria from the ethnomedicinal *Mussaenda roxburghii* (Akshap) of Eastern Himalayan Province, India. Adv. Biol..

[86] Lorck H (1948). Production of hydrocyanic acid by bacteria. Physiol. Plant..

[87] Sultan A, Nabiel Y (2019). Tube method and Congo red agar versus tissue culture plate method for detection of biofilm production by uropathogens isolated from midstream urine: Which one could be better?. Afr. J. Clin. Exp. Microbiol..

[88] Antognoni F, Mandrioli R, Potente G, Taneyo Saa DL, Gianotti A (2019). Changes in carotenoids, phenolic acids and antioxidant capacity in bread wheat doughs fermented with different lactic acid bacteria strains. Food Chem..

[89] Copaciu F, Opriş O, Niinemets Ü, Copolovici L (2016). Toxic influence of key organic soil pollutants on the total flavonoid content in wheat leaves. Water Air Soil Pollut..

[90] Shah SH, Houborg R, McCabe MF (2017). Response of chlorophyll, carotenoid and SPAD-502 measurement to salinity and nutrient stress in wheat (*Triticum aestivum* L.). Agronomy.

[91] Wright ES, Yilmaz LS, Noguera DR (2012). DECIPHER, a search-based approach to chimera identification for 16S rRNA sequences. Appl. Environ. Microbiol..

[92] Franck AR (2012). Guide to agave, cinnamomum, corymbia, eucalyptus, pandanus, and sansevieria in the flora of Florida. Phytoneuron.

[93] Garden, M. B. *Agave desmetiana*, https://www.missouribotanicalgarden.org/PlantFinder/plantfindersearch.aspx.

[94] Mbarki, S. *et al.* in *Salinity responses and tolerance in plants* (Eds. V. Kumar, Wani, S., Suprasanna, P., Tran, LS.) pp. 85–136, Vol. 1 (Springer, Cham, 2018). 10.1007/978-3-319-75671-4_4.

[95] Huang W-Y, Cai Y-Z, Xing J, Corke H, Sun M (2007). A potential antioxidant resource: Endophytic fungi from medicinal plants. Econ. Bot..

[96] Nakabayashi R (2014). Enhancement of oxidative and drought tolerance in *Arabidopsis* by overaccumulation of antioxidant flavonoids. Plant J. Cell Mol. Biol..

[97] El Sabagh A (2021). Salinity stress in wheat (*Triticum aestivum* L.) in the changing climate: Adaptation and Management Strategies. Front. Agronomy.

[98] Sahab S (2021). Potential risk assessment of soil salinity to agroecosystem sustainability: Current status and management strategies. Sci. Total Environ..

[99] Daliakopoulos IN (2016). The threat of soil salinity: A European scale review. Sci. Total Environ..

[100] Nazari Nooghabi S (2022). Social, economic and environmental vulnerability: The case of wheat farmers in Northeast Iran. Sci. Total Environ..

[101] Lozo J (2023). Rhizosphere microbiomes of resurrection plants *Ramonda serbica* and *R. nathaliae*: Comparative analysis and search for bacteria mitigating drought stress in wheat (*Triticum aestivum* L.). World J. Microbiol. Biotechnol..

[102] Pramanic A, Sharma S, Dhanorkar M, Prakash O, Singh P (2023). Endophytic microbiota of floating aquatic plants: Recent developments and environmental prospects. World J. Microbiol. Biotechnol..

[103] Akhtar N, Wani AK, Dhanjal DS, Mukherjee S (2022). Insights into the beneficial roles of dark septate endophytes in plants under challenging environment: Resilience to biotic and abiotic stresses. World J. Microbiol. Biotechnol..

[104] Hagaggi NSA, Abdul-Raouf UM (2022). Drought-tolerant *Sphingobacterium changzhouense* Alv associated with Aloe vera mediates drought tolerance in maize (*Zea mays*). World J. Microbiol. Biotechnol..

[105] Sharma M, Sood G, Chauhan A (2021). Bioprospecting beneficial endophytic bacterial communities associated with *Rosmarinus officinalis* for sustaining plant health and productivity. World J. Microbiol. Biotechnol..

[106] Kaur J (2023). Reactive Black-5, Congo Red and Methyl Orange: Chemical degradation of Azo-Dyes by *Agrobacterium*. Water.

[107] Vinayak, A., Mudgal, G., Sharma, S. & Singh, G. B. in *Advances in Probiotics for Sustainable Food and Medicine* (Eds. Gunjan Goel & Ashok Kumar) pp. 63–82 (Springer, Singapore, 2021). 10.1007/978-981-15-6795-7_4.

[108] Mudgal G, Mudgal B (2011). Evidence for unusual choice of host and haustoria by *Dendrophthoe falcata* (L.f) Ettingsh, a leafy mistletoe. Arch. Phytopathol. Plant Prot..

[109] Kaur J (2023). GC-MS validated phytochemical up-leveling with in vitro-raised *Sansevieria trifasciata* [Prain]: The Mother in Law’s tongue gets more antibacterial. Curr. Plant Biol..

[110] Ash C, Farrow JAE, Wallbanks S, Collins MD (1991). Phylogenetic heterogeneity of the genus *Bacillus* revealed by comparative analysis of small-subunit-ribosomal RNA sequences. Lett. Appl. Microbiol..

[111] Gupta RS, Patel S, Saini N, Chen S (2020). Erratum: Robust demarcation of seventeen distinct *Bacillus* species clades, proposed as novel Bacillaceae genera, by phylogenomics and comparative genomic analyses: description of *Robertmurraya kyonggiensis* sp. nov. and proposal for emended genus *Bacillus* limiting it only to the members of the subtilis and cereus clades of species. Int. J. Syst. Evolut. Microbiol..

[112] Patel S, Gupta RS (2020). A phylogenomic and comparative genomic framework for resolving the polyphyly of the genus *Bacillus*: Proposal for six new genera of *Bacillus* species, *Peribacillus* gen. nov., *Cytobacillus* gen. nov., *Mesobacillus* gen. nov., *Neobacillus* gen. nov., *Metabacillus* gen. nov. and *Alkalihalobacillus* gen. nov. Int. J. Syst. Evolut. Microbiol..

[113] Bhattacharyya C (2017). Genome-guided insights into the plant growth promotion capabilities of the physiologically versatile *Bacillus aryabhattai* strain AB211. Front. Microbiol..

[114] Ghosh PK (2018). The role of arsenic resistant *Bacillus aryabhattai* MCC3374 in promotion of rice seedlings growth and alleviation of arsenic phytotoxicity. Chemosphere.

[115] Kulkova I, Dobrzyński J, Kowalczyk P, Bełżecki G, Kramkowski K (2023). Plant growth promotion using *Bacillus cereus*. Int. J. Mol. Sci..

[116] Zhou H (2021). Efficacy of plant growth-promoting bacteria *Bacillus cereus* YN917 for biocontrol of rice blast. Front. Microbiol..

[117] Zhao J-L, Zhou L-G, Wu J-Y (2010). Promotion of *Salvia miltiorrhiza* hairy root growth and tanshinone production by polysaccharide–protein fractions of plant growth-promoting rhizobacterium *Bacillus cereus*. Process Biochem..

[118] Jetiyanon K, Wittaya-Areekul S, Plianbangchang P (2008). Film coating of seeds with *Bacillus cereus* RS87 spores for early plant growth enhancement. Can. J. Microbiol..

[119] Ku Y (2018). Root colonization and growth promotion of soybean, wheat and Chinese cabbage by *Bacillus cereus* YL6. PLoS ONE.

[120] Kumar P (2020). Effect of silver nanoparticles and *Bacillus cereus* LPR2 on the growth of *Zea mays*. Sci. Rep..

[121] Patani A (2024). Recent advances in *Bacillus*-mediated plant growth enhancement: A paradigm shift in redefining crop resilience. World J. Microbiol. Biotechnol..

[122] Shahid M (2022). Stress-tolerant endophytic isolate *Priestia aryabhattai* BPR-9 modulates physio-biochemical mechanisms in wheat (*Triticum aestivum* L.) for enhanced salt tolerance. Int. J. Environ. Res. Public Health.

[123] Zelaya-Molina LX (2023). Plant growth-promoting and heavy metal-resistant Priestia and *Bacillus* strains associated with pioneer plants from mine tailings. Arch. Microbiol..

[124] Abiala M, Sadhukhan A, Sahoo L (2023). Isolation and characterization of stress-tolerant priestia species from cowpea rhizosphere under drought and nutrient deficit conditions. Curr. Microbiol..

[125] Moturu US (2023). Investigating the diversity of bacterial endophytes in maize and their plant growth-promoting attributes. Folia Microbiol..

[126] Li Q (2022). A plant growth-promoting bacteria *Priestia megaterium* JR48 induces plant resistance to the crucifer black rot via a salicylic acid-dependent signaling pathway. Front. Plant Sci..

[127] Deng C (2022). Molecular mechanisms of plant growth promotion for methylotrophic *Bacillus aryabhattai* LAD. Front. Microbiol..

[128] Nobel PS, Berry WL (1985). Element responses of agaves. J. Appl. Ecol..

[129] Miyamoto, S. *Salt tolerance of landscape plants common to the southwest*, http://hdl.handle.net/1969.1/86110 (2008).

[130] Peña-Valdivia CB, Sánchez-Urdaneta AB (2009). Effects of substrate water potential in root growth of *Agave salmiana* Otto ex Salm-Dyck seedlings. Biol. Res..

[131] Schuch, U. K. & Kelly, J. J. (College of Agriculture and Life Sciences, University of Arizona (Tucson, AZ), 2008). http://hdl.handle.net/10150/216639.

[132] Srinivasa C (2022). Plants and endophytes—A partnership for the coumarin production through the microbial systems. Mycology.

[133] Mahmud FMA (2023). Effects of halotolerant rhizobacteria on rice seedlings under salinity stress. Sci. Total Environ..

