# Supplementary material for: Novel Bacillus and Prestia isolates from Dwarf century plant enhance crop yield and salinity tolerance
Source: Sci Rep. 2024 Jun 25;14:14645. doi: 10.1038/s41598-024-65632-x (PMC11199671; doi:10.1038/s41598-024-65632-x)
Supplement: Supplementary file 1 — Supplementary Information. [file 41598_2024_65632_MOESM1_ESM.docx]

Novel *Bacillus* and *Prestia* Isolates from Dwarf Century Plant Enhance Crop Yield and Salinity Tolerance

# Sanjoy Kumar Dhar^1+^, Jaspreet Kaur^1+^, Gajendra Bahadur Singh^1^, Arjun Chauhan^2^, Jeewan Tamang^3,4^, Nikita Lakhara^1^, Lyudmila Asyakina^5^, Victor Atuchin^6,7,8,9^, Gaurav Mudgal^1, 10^*, Gholamreza Abdi^11^*

^1^ University Institute of Biotechnology, Chandigarh University, Mohali, Punjab-140413, India

^2^ Department of Biotechnology, Institute of Applied Sciences & Humanities, GLA University, Mathura 281406, Uttar Pradesh, India

^3^ University Institute of Agricultural Sciences, Chandigarh University, Mohali, Punjab-140413, India

^4^ Khaniyabas Rural Municipality, Dhading, Province 3, Bagmati Zone 45100, Nepal

_5_ Laboratory for Phytoremediation of Technogenically Disturbed Ecosystems, Kemerovo State University, Krasnaya Street, 6, 650000 Kemerovo, Russia

^6^ Laboratory of Optical Materials and Structures, Institute of Semiconductor Physics, SB RAS, Novosibirsk 630090, Russia

^7^ Research and Development Department, Kemerovo State University, Kemerovo 650000, Russia

^8^ Department of Industrial Machinery Design, Novosibirsk State Technical University, Novosibirsk 630073, Russia

^9^ R&D Center “Advanced Electronic Technologies”, Tomsk State University, Tomsk 634034, Russia

^10^ Center for Waste Management and Renewable Energy, Saveetha Dental College and Hospitals, Saveetha Institute of Medical and Technical Sciences, Saveetha University, Chennai 600077, India

^11^Department of Biotechnology, Persian Gulf Research Institute, Persian Gulf University, Bushehr 75169, Iran

+ Authors with equal contribution and share the first authorship.

* Corresponding authors

**Supplementary Table 1: Biochemical, and morphological profiles of the ADJ isolates.**

**Supplementary Table 2. Antibiotic tolerance of ADJ1 and ADJ6** **isolates.**

**Supplementary Figure. 1 SEM details over the ADJ1 and ADJ6.**

**Supplementary Figure. 2 Growth and yield parameters of wheat treated with ADJ1 and ADJ6 compared to untreated controls after harvested field trials.**

**Supplementary Figure. 3 Germination extent, seedling morphometrics, and physiological growth of ADJ endophyte-treated wheat under salinity stress.**

**Supplementary Table 1: Biochemical, and morphological profiles of the ADJ isolates.**

| ***Assay classes*** | ***Activity/assays*** | ***Characteristics*** | |  |
| --- | --- | --- | --- | --- |
| ***Morphology and growth responses*** | | ADJ1 | ADJ6 |  |
|  | Gram reaction | +ve | +ve |  |
|  | Shape in LM and SEM | Rod-shaped | Rod-shaped |  |
|  | Colony on NA | Smooth, irregularly shaped, pale white | Smooth, irregularly shaped, pale white |  |
|  | Colony on MSA | Smooth and white | Mucoid and white |  |
|  | Colony on LBA | Smooth, irregularly shaped, white | Clustered, irregularly shaped, yellowish-white |  |
| ***Standard biochemical responses*** | | |  | |
|  | Catalase test | + | + | |
|  | Methyl red | - | + | |
|  | Indole test | - | + | |
|  | Citrate utilization | + | + | |
|  | Voges Proskauer | - | - | |
|  | Starch hydrolysis | - | + | |
|  | Urease test | + | + | |
|  | Oxidase test | + | + | |
|  | Motility test | + | + | |
|  | Hydrogen sulfide test | + | + | |
|  | Tween-20 hydrolysis | - | - | |
|  | Tween-80 hydrolysis | - | - | |
|  |  |  |  | |
| ***Carbohydrate utilization*** | | |  | |
|  | Glucose | **-** | **+** | |
|  | Sucrose | - | + | |
|  | Starch | - | + | |
|  | Mannitol | + | + | |
|  | Lactose | - | + | |
|  | Dextrose | - | + | |
|  | Gelatin | - | + | |
|  | Adonitol | - | - | |
|  | Arabinose | + | - | |
|  | Sorbitol | - | - | |
|  | Rhamnose | + | - | |
| ***Growth in NaCl*** | | |  | |
|  | 1% | + | + | |
|  | 2% | + | + | |
|  | 3% | + | + | |
|  | 4% | + | + | |
|  | 5% | + | + | |
|  | 6% | + | + | |
|  | 8% | - | - | |
| ***Enzyme activities*** | |  |  | |
|  | Cellulase | + | + | |
|  | Protease | - | - | |
|  | Lipase | - | - | |
|  | Pectinase | + | + | |
|  | Amylase | + | + | |

**Supplementary Table 2. Antibiotic tolerance of ADJ1 and ADJ6** **isolates.** Disc size= 6mm; S=Less susceptibility (7-10mm); S^+^=Susceptibility (11-20mm); S^++^= High susceptibility (21-30mm); S^+++^= Extreme susceptibility (31-40mm); R=Resistant (0 mm). All antibiotics were purchased from Himedia (Mumbai, India) with indicated catalog numbers (*Cat#*) in the first column.

| Cat# | Antibiotic (Concentration) | **ADJ1**  **ADJ6** | | | |
| --- | --- | --- | --- | --- | --- |
|  |  | Inhibition Response Inhibition Response  Zone (mm) Zone (mm) | | | |
| SD076 | Amoxicilin (10µg) | 13.00±0.50 | S+ | 9.00±0.50 | S |
| SD184 | Norfloxacin (5µg) | 22.00±0.25 | S++ | 48.33±0.50 | S+++ |
| SD133 | Tetracycline (30µg) | 9.00±0.25 | S | 24.66±0.25 | S++ |
| SD060 | Ciprofloxacin (30µg) | 28.66±0.25 | S++ | 31.66±0.50 | S+++ |
| SD016 | Gentamicin (50µg) | 35.33±0.50 | S+++ | 29.00±0.25 | S++ |
| SD031 | Streptomycin (10µg) | 20.00±0.50 | S+ | 16.33±0.50 | S+ |
| SD002 | Ampicillin (10µg) | 0 | Resistant | 0 | Resistant |
| SD044 | Tobramycin (10µg) | 29.66±0.40 | S++ | 30.33±0.50 | S++ |
| SD039 | Trimethoprim (5µg) | 33.66±0.25 | S+++ | 31.33±0.25 | S+++ |
| SD028 | Penicillin (10 units) | 0 | Resistant | 20.67±0.25 | S+ |
| SD006 | Chloramphenicol (10µg) | 30.00±0.33 | S++ | 29.67±0.25 | S++ |
| EM018 | Clarithromycin (15 µg) | 24.00±0.25 | S++ | 27.67±0.50 | S++ |
| SD731 | Neomycin (10µg) | 24.00±0.50 | S++ | 24.50±0.25 | S++ |
| SD040 | Cefotaxime (30µg) | 21.33±0.25 | S++ | 33.67±0.25 | S+++ |
| SD219 | Cefepime (30µg) | 0 | Resistant | 13.67±0.50 | S+ |
| SD069 | Ofloxacin (5 µg) | 30.00±0.50 | S++ | 22.67±0.50 | S++ |


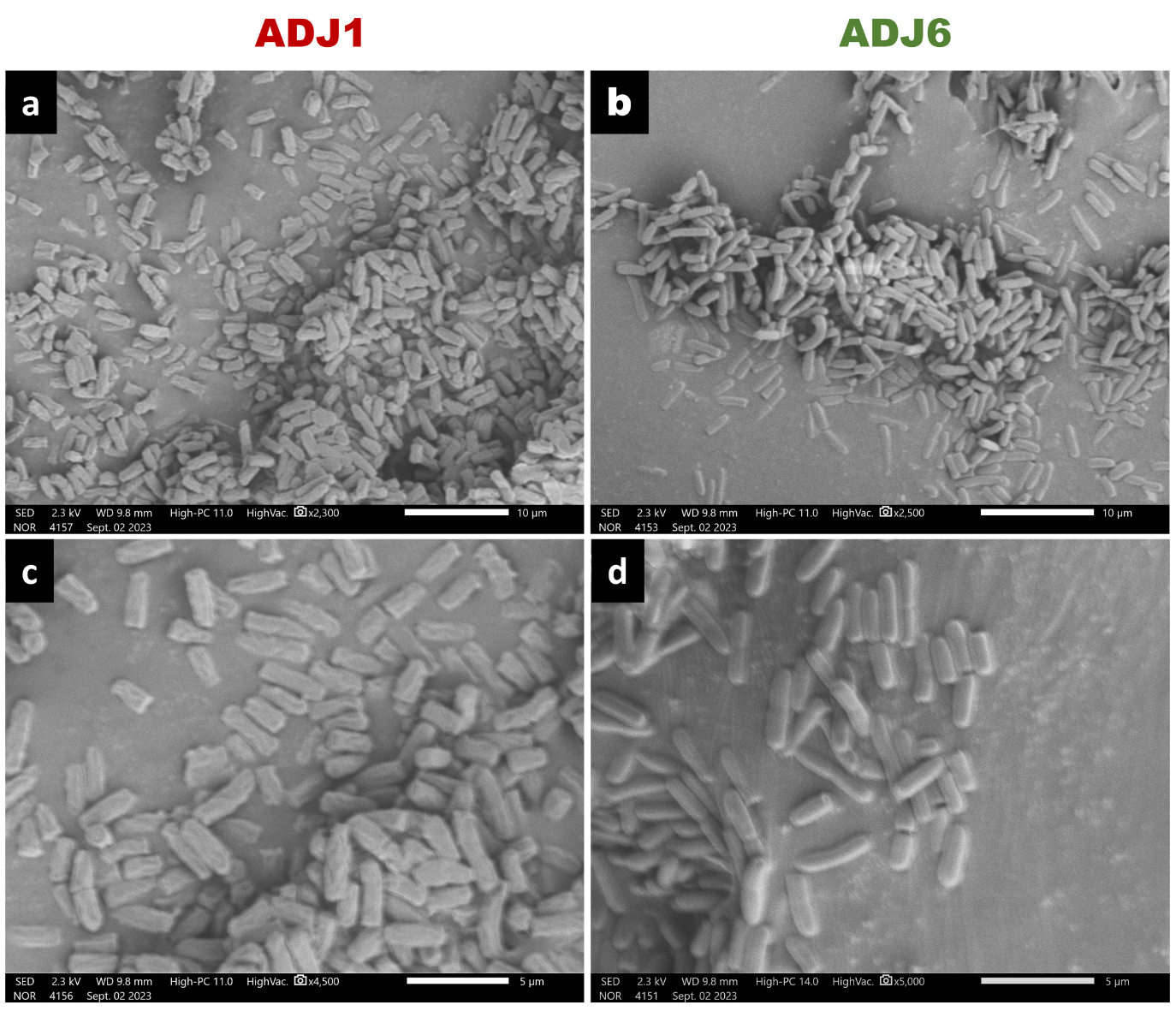


**Supplementary Figure. 1 SEM details over the ADJ1 and ADJ6.** In figure panels **a**, **c**, morphological details of ADJ1; in panels **b**, **d**, the details of ADJ6. Note the different physical features of the two ADJ-isolated bacteria.


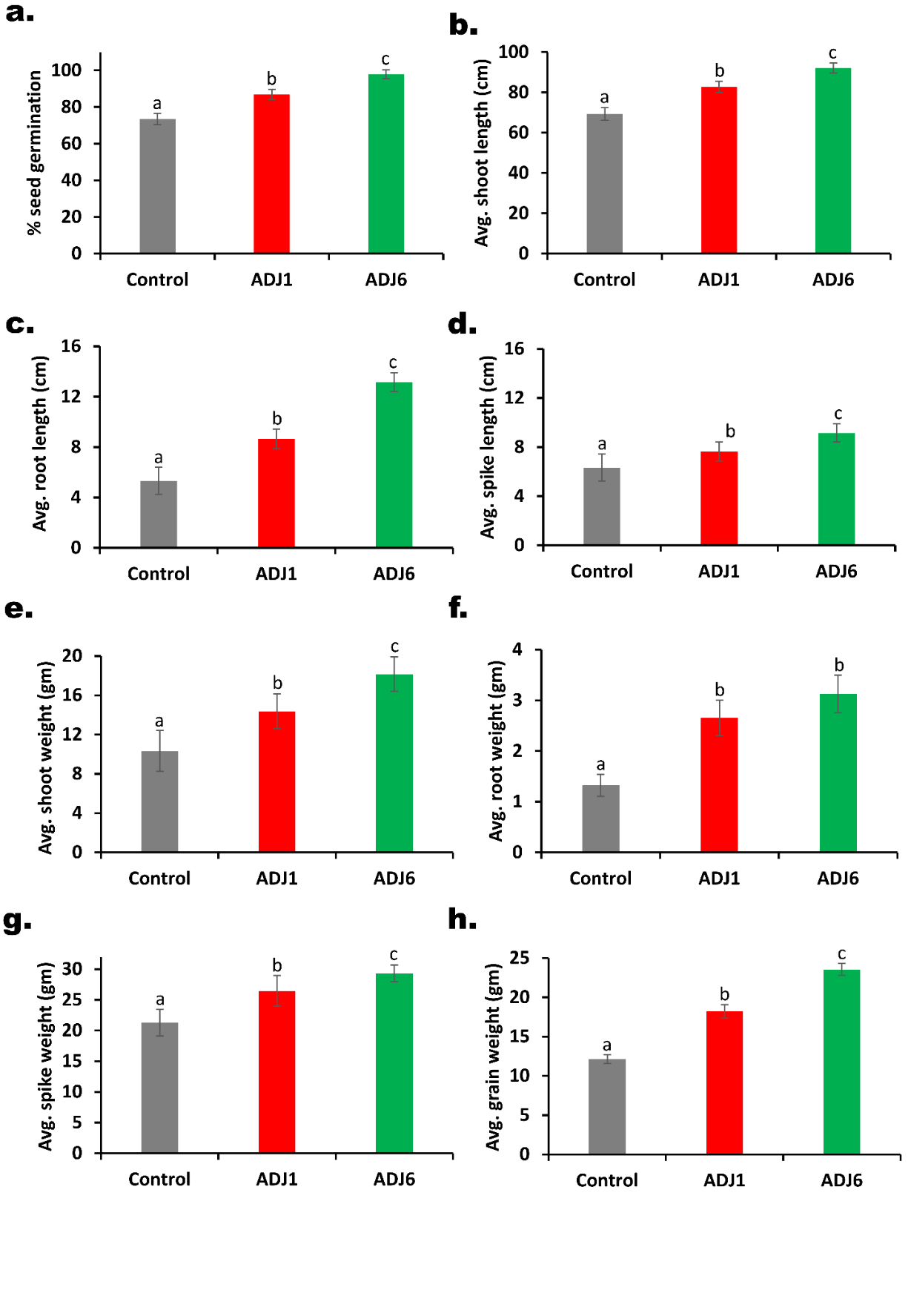


**Supplementary Figure. 2 Growth and yield parameters of wheat treated with ADJ1 and ADJ6 compared to untreated controls after harvested field trials.** The ADJ1/ADJ6 treated wheat showed enhanced a) Seed germination percentage; b) Shoot length; c) Root length; d) Spike length; e) Root weight; f) Shoot weight; g) Spike weight; and h) Grain weight. The ADJ1 and ADJ6 wheat had higher values than control wheat across all measurements. Field trials were performed in triplicate with 3 replicate plots per trial set up in a Completely Randomized Design. The graphed values represent the overall means of the 3 independent trials, with error bars indicating standard deviations. Also, note the average values measured for each group are denoted with different superscript letters, indicating statistically significant differences between the groups as determined by Duncan's multiple range test at a 5% significance level (p < 0.05).


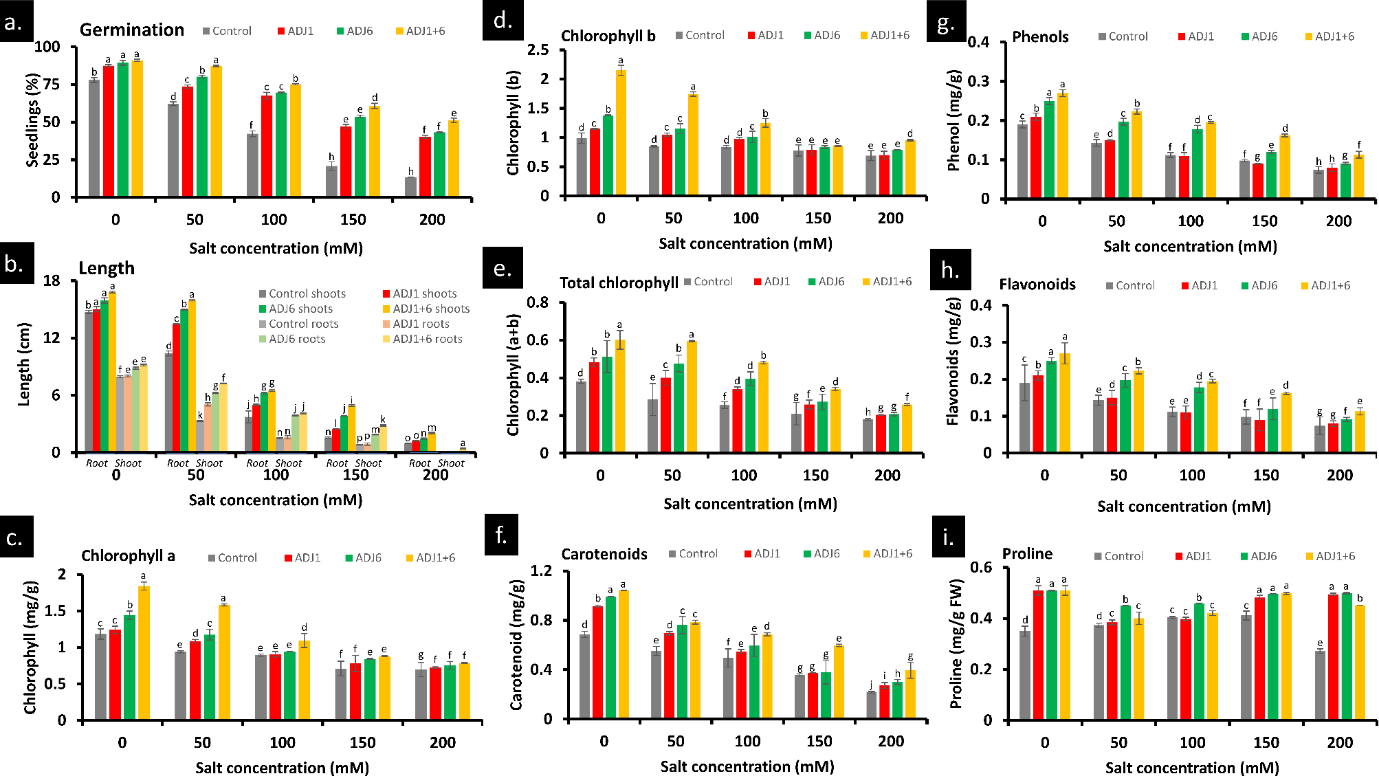


**Supplementary Figure. 3 Germination extent, seedling morphometrics, and physiological growth of ADJ endophyte-treated wheat under salinity stress.** In **a**, percentage germination; **b**, Shoot and root morphometrics; and in **c-i**, various physiological parameters adjudged in control untreated wheat seeds versus bacterized seed lots with either ADJ1, ADJ6 and a combination of ADJ1 plus ADJ6 (=ADJ1+6) in the effect from various salinity concentration in the germination medium. Data values depict the means of at least three independent trials with their standard deviations shown with error bars. Values for total phenols depict gallic acid equivalents, and for flavonoids depict quercetin equivalents. Also note that the average values measured for each group are denoted with different superscript letters, indicating statistically significant differences between the groups as determined by Duncan's multiple range test at a 5% significance level (p < 0.05).
